# A Supported Bismuth Halide Perovskite Photocatalyst for Selective Aliphatic and Aromatic C–H Bond Activation

**DOI:** 10.1002/anie.201915034

**Published:** 2020-01-23

**Authors:** Yitao Dai, Corentin Poidevin, Cristina Ochoa‐Hernández, Alexander A. Auer, Harun Tüysüz

**Affiliations:** ^1^ Max-Planck-Institut für Kohlenforschung Kaiser-Wilhelm-Platz 1 45470 Mülheim an der Ruhr Germany

**Keywords:** bismuth, nanoparticles, perovskites, photocatalysis, SBA-15 silica

## Abstract

Direct selective oxidation of hydrocarbons to oxygenates by O_2_ is challenging. Catalysts are limited by the low activity and narrow application scope, and the main focus is on active C−H bonds at benzylic positions. In this work, stable, lead‐free, Cs_3_Bi_2_Br_9_ halide perovskites are integrated within the pore channels of mesoporous SBA‐15 silica and demonstrate their photocatalytic potentials for C−H bond activation. The composite photocatalysts can effectively oxidize hydrocarbons (C_5_ to C_16_ including aromatic and aliphatic alkanes) with a conversion rate up to 32900 μmol g_cat_^−1^ h^−1^ and excellent selectivity (>99 %) towards aldehydes and ketones under visible‐light irradiation. Isotopic labeling, in situ spectroscopic studies, and DFT calculations reveal that well‐dispersed small perovskite nanoparticles (2–5 nm) possess enhanced electron–hole separation and a close contact with hydrocarbons that facilitates C(sp^3^)−H bond activation by photoinduced charges.

## Graphical abstract

**Better with bismuth**: A range of mesoporous‐silica‐supported small bismuth halide perovskite nanoparticles were prepared. They were found to be very promising photocatalysts for selective aromatic and aliphatic C−H bond activation under visible‐light illumination.

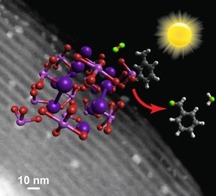

## Introduction

The selective oxidation of sp^3^ C−H bonds using O_2_ to valuable oxygenated products is one of the most important reactions in the chemical industry for fine chemicals and pharmaceuticals production.^1^ In comparison with the partial oxidation of active and expensive alcohol substrates,^2^ the direct conversion of hydrocarbons (for example, aliphatic alkanes) in air to commercial oxygenates remains challenging. This is due to the high bond dissociation energy of C(sp^3^)−H bonds (70–130 kcal mol^−1^) and their unfavorable adsorption.1b, 3 The activation of C(sp^3^)−H bonds generally demands specific expensive metal complexes or aggressive oxidants with harsh reaction conditions (high pressure and temperature).^4^ Additionally, in some cases the conversion has to be suppressed (<15 %) to avoid poor selectivity owing to over‐oxidation products like CO and CO_2_.^5^

Photocatalytic organic synthesis is a promising and green approach that is performed under mild conditions with solar energy as driving force.^6^ To date, several photocatalytic systems have been reported for aromatic oxidations.^7^ For example, CdS,7c Bi_2_WO_6_/BiOCl,7f and TiO_2_/Bi_2_MO_6_ 7g have activated benzylic C−H bonds under visible light illumination. Very recently, lead‐based perovskite NiO_*x*_/FAPbBr_3_/TiO_2_ composite was reported to activate C−H bond under solar light irradiation; however, the production rate dropped tenfold when only visible light was used as light source.7i The photocatalysts developed so far are limited by low efficiencies (the highest reported conversion rate is 4388 μmol g_cat_^−1^ h^−1^ for toluene oxidation)7f and narrow substrate scopes, mainly focusing on C−H bonds at benzylic or allylic positions. Thus, there is a need to design more effective photocatalysts for selective C−H bond activation.

Among narrow‐band gap semiconductor materials, all‐inorganic halide perovskites (ABX_3_ with A=Rb, Cs; B=Ge, Pb, Sn; and X=Cl, Br, I) have gained increasing attention owing to their unique photovoltaic and optoelectronic properties.^8^ With the rapid growth in solar cells, many different novel research avenues on halide perovskites have emerged. In contrast, they are hardly explored in heterogeneous catalysis.^9^ Following the first report using MAPbI_3_ for photocatalytic hydrogen evolution,^10^ lately toxic lead‐based perovskites (for example, CsPbBr_3_ and CsPbI_3_) have been used for photocatalytic reactions such as dye degradation,^11^ CO_2_ reduction,8b, 12 and various organic transformations including C(sp^3^)−H bond activation,7i alcohol oxidation,^13^ aldehyde alkylation,^14^ thiol reaction,^15^ C−C cleavage, and dehydrogenation.^16^ Moreover, CsPbBr_3_ nanocrystals have successfully driven other organic synthesis such as C−C, C−O, and C−N bond‐forming reactions.^17^ In comparison, cheap, low‐toxic, and air‐stable bismuth‐based counterparts such as Cs_3_Bi_2_X_9_ 18 and Cs_2_AgBiX_6_ 19 showed a few applications in photocatalysis (CO_2_ reduction,19e ring‐opening reactions,^20^ and dye degradation^21^).

Herein, we report a novel class of ordered mesoporous SBA‐15 silica supported halide perovskite photocatalysts. Halide perovskite nanoparticles with a size of 2–5 nm are confined within pore channels of SBA‐15, which provides large numbers of catalytically active centers. Consequently, SBA‐15 supported Cs_3_Bi_2_Br_9_ nanoparticles show outstanding catalytic performance for selective aliphatic and aromatic C(sp^3^)−H bond activation under visible light irradiation. Electronic structure calculations using density functional theory (DFT) indicate that clusters like Cs_12_Bi_14_Br_54_ with a size of a few nanometers are stable and have bismuth‐rich surfaces. The optimized photocatalyst, 10 wt % Cs_3_Bi_2_Br_9_/SBA‐15, displays a high conversion rate of up to ca. 32 900 μmol g_cat_^−1^ h^−1^, going beyond state‐of‐the‐art catalysts. A quantum efficiency (QE) of 11 % is achieved under 1 sun illumination. By using toluene oxidation as a model reaction, our experimental, spectroscopic, and computational studies reveal that supported perovskite nanoparticles show enhanced charge separation and close interactions with hydrocarbons that promote C−H bond activation.

## Results and Discussion

### Catalyst Characterization

**Figure 1:**
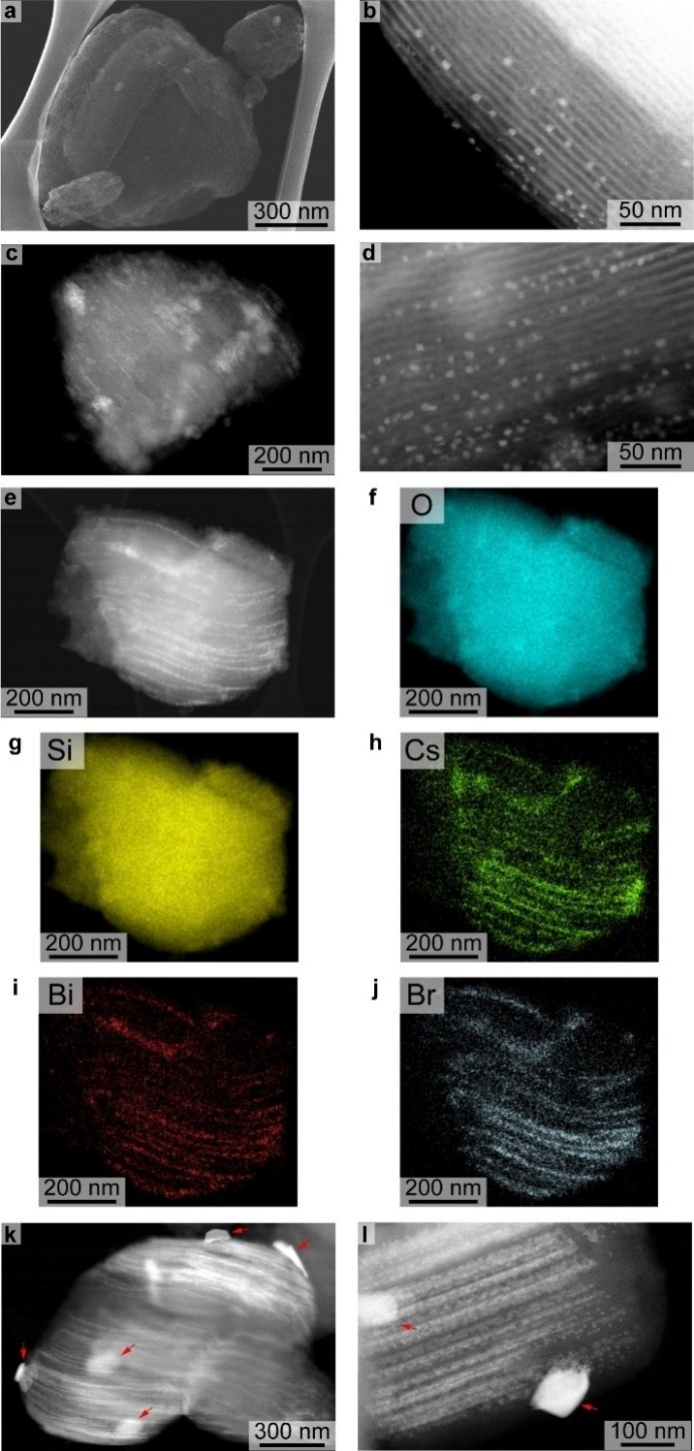
STEM analysis of Cs_3_Bi_2_Br_9_/SBA‐15 samples with different loadings of perovskite nanoparticles. a) SE‐STEM and b) HAADF‐STEM images of 5 wt % loading. c),d) HAADF‐STEM images of 10 wt % loading. e)–j) HAADF‐STEM image and element mapping analysis of 20 wt % loading. k),l) HAADF‐STEM images of 40 wt % loading (large Cs_3_Bi_2_Br_9_ particles are indicated by red arrows).

The halide perovskite Cs_3_Bi_2_Br_9_ nanoparticles were formed within the mesopores of SBA‐15 silica with different loadings (calculated 5, 10, 20, 40 wt %; Supporting Information, Figure S1 d, and Table S1 for elemental analysis) by the incipient wetness impregnation (IWI) method.^22^ For the Cs_3_Bi_2_Br_9_/SBA‐15 sample with 5 wt % loading of perovskite nanoparticles, scanning transmission electron microscopy (STEM) in secondary electron (SE) mode (Figure 1 a) shows a typical morphology of SBA‐15.^23^ As shown in Figure 1 b, high‐angle annular dark‐field mode (HAADF) STEM clearly demonstrates the successful confinement of small Cs_3_Bi_2_Br_9_ nanoparticles with a size of 2–5 nm in the ordered channels (pore size ca. 8 nm) of SBA‐15 silica, and nanoparticles are well‐separated. When the loading is increased to 10 wt %, the high dispersion of small perovskite nanoparticles is still maintained as illustrated by Figure 1 c,d. A higher loading of 20 wt % makes the pore filling by Cs_3_Bi_2_Br_9_ much more compact, leading to poor utilization of available space in the support matrix and active sites on the perovskite surface. Some parts of the channels of SBA‐15 were completely filled, resulting in more aggregated perovskite nanoparticles, as seen in Figure 1 e. Meanwhile, the element mapping results (Figure 1 f–j) demonstrate that the material confined in SBA‐15 channels is halide perovskite, while its crystallinity was confirmed by powder X‐ray diffraction (XRD) (Supporting Information, Figure S1 a,b). At the highest loading of 40 wt %, formation of bulk halide perovskites on the structure of SBA‐15 silica is observed along with the pore confined nanoparticles (Figure 1 k,l). With the IWI method, a very homogenous distribution of perovskite crystals over mesoporous silica cannot be achieved, thus pores of SBA‐15 stay unfilled in some areas (Supporting Information, Figure S1 c).

In agreement with the electron microscopy analysis, the XRD results (Supporting Information, Figure S1 a,b) show that the samples with lower loadings (5 wt % and 10 wt %) presented weak and broad characteristic diffraction peaks of Cs_3_Bi_2_Br_9_, indicating small perovskite nanoparticles. SBA‐15 silica kept its ordered structure even at a high perovskite loading of 40 wt %. Additionally, based on the N_2_‐physisorption analysis (Supporting Information, Figure S2), the samples with lower perovskite loadings (5 wt % and 10 wt %) have similar pore volumes (1.1 cm^3^ g^−1^) and surface areas (802 and 767 m^2^ g^−1^, respectively). Increasing the loading amount to 20 wt % and 40 wt % causes a drop in pore volumes (0.9 and 0.6 cm^3^ g^−1^) and surface areas (677 and 473 m^2^ g^−1^) for the composite materials owing to the gradual blockage of mesopores by aggregated perovskite particles, as seen in STEM images in Figure 1.

**Figure 2:**
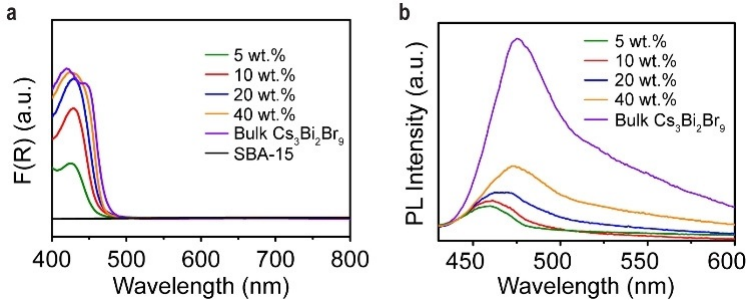
UV/Vis DRS and PL spectra of Cs_3_Bi_2_Br_9_/SBA‐15 samples. a) Light absorption of Cs_3_Bi_2_Br_9_/SBA‐15 with different loadings, bare SBA‐15 and bulk Cs_3_Bi_2_Br_9_. b) PL spectra of supported and bulk Cs_3_Bi_2_Br_9_.

The optical properties of as‐prepared Cs_3_Bi_2_Br_9_/SBA‐15 were further characterized by UV/Vis diffuse reflectance (DRS) and photoluminescence (PL) spectroscopy. Figure 2 a shows that all samples have visible light absorption extended to the wavelength of 500 nm. As expected, increase of loading enhances light absorption since Cs_3_Bi_2_Br_9_ has the capability to absorb visible light as indicated by the DRS results of bulk Cs_3_Bi_2_Br_9_ and bare SBA‐15 (Figure 2 a). The optical band gap (*E*_g_) increased gradually as the loading decreased due to the quantum confinement effect for smaller nanoparticles (Supporting Information, Figure S4 a).^24^ The PL spectra of supported samples (Figure 2 b) clearly show a red‐shift of main emission peaks as the loading increased, matching well with the gradually decreased *E*_g_. Moreover, the samples with lower loadings (5 and 10 wt %) showed a much lower PL intensity, indicating the suppressed radiative recombination of photoinduced electron–hole pairs.12a This may originate from the smaller perovskite nanoparticles with shorter diffusion distance of charges in excited state.^25^ Thus, Cs_3_Bi_2_Br_9_/SBA‐15 samples with lower loadings seems to have better charge separation. After adsorption of hydrocarbons (for example, toluene), the supported samples with low perovskite loadings (5 and 10 wt %) display an additional absorption band in the visible range (from 500 to 750 nm; Supporting Information, Figure S4 b), which indicates strong interactions between aromatics and Cs_3_Bi_2_Br_9_/SBA‐15 composite with Bi atoms as Lewis acid sites (Supporting Information, Figure S3).^20^ These types of interactions might promote the catalytic efficiency of the photocatalysts. The FTIR spectroscopy showed no strong intramolecular interactions between hydrocarbons and composite material (Supporting Information, Figure S4 g).^26^

### Photocatalytic Oxidation of Aliphatic and Aromatic Hydrocarbons

**Figure 3:**
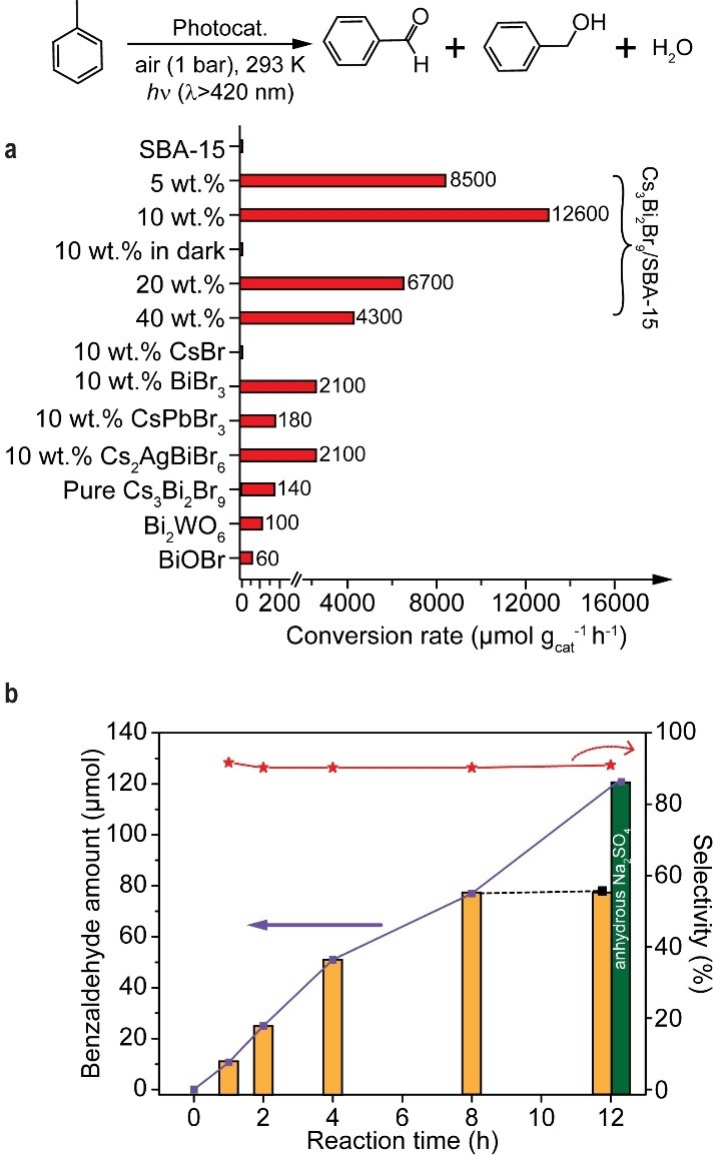
Photocatalytic oxidation of toluene over diverse catalysts. a) Toluene conversion rate over different samples. Conditions: 1 bar air, 293 K, 5 mL toluene, 10 mg photocatalyst, 2 h irradiation (≥420 nm). For all supported samples, SBA‐15 was used as support and the effective catalyst mass was the weight of loaded halide perovskite phase. b) Time dependence of toluene photo‐oxidation over 10 wt % Cs_3_Bi_2_Br_9_/SBA‐15. The green bar indicates the result upon adding anhydrous Na_2_SO_4_ in the beginning of the reaction.

After catalyst characterizations, we employed as‐prepared Cs_3_Bi_2_Br_9_/SBA‐15 as photocatalysts to activate C(sp^3^)−H bonds of hydrocarbons in air under visible light irradiation. By using toluene as a model substrate, the catalytic performance of several photocatalysts were systematically evaluated in a home‐made setup (Supporting Information, Figure S5). The control experiments show that the absence of light or the halide perovskite phase (no catalyst or bare SBA‐15 support) led to no production of oxygenates from toluene. As shown in Figure 3 a, for the supported Cs_3_Bi_2_Br_9_/SBA‐15 photocatalysts, the sample with a loading of 10 wt % has the highest conversion rate of 12 600 μmol g_cat_^−1^ h^−1^ and high selectivity of 90 % towards benzaldehyde (benzyl alcohol as the main by‐product). Meanwhile, no increase of CO/CO_2_ was detected in the gas phase using gas chromatography (GC) and mass spectrometry (MS; Supporting Information, Figure S6). In contrast, the 5 wt % Cs_3_Bi_2_Br_9_/SBA‐15 photocatalyst showed lower activity owing to a lower number of active centers and weaker light absorption (Figure 2 a). In the cases of higher loadings (20 and 40 wt %) and bulk Cs_3_Bi_2_Br_9_, the dramatic drop of activities (down to 140 μmol g_cat_^−1^ h^−1^) was observed mainly due to limited accessible active sites and serious charge recombination with aggregated or large nanoparticles.

The catalytic survey of 40 wt % supported and bulk perovskite samples suggests that the specific interactions with toluene indicated by UV/Vis DRS analysis are not essential to initiate C−H bond activation. Meanwhile, the toluene conversion rate could be promoted when the strong interactions are present (Supporting Information, Figure S4 b). This correlation also applies for other halide perovskites (for example, Cs_2_AgBiBr_6_ and CsPbBr_3_). Although supported Cs_2_AgBiBr_6_ and CsPbBr_3_ nanoparticles on SBA‐15 can absorb more photons due to smaller band gaps (2.28 and 2.33 eV respectively), they showed lower photocatalytic activity (2100 and 200 μmol g_cat_^−1^ h^−1^ respectively), which corresponds well with their weaker interactions with toluene (Supporting Information, Figure S4 d). Furthermore, the CsBr/SBA‐15 sample presented no activity, while the BiBr_3_ counterpart was active for the activation of C−H bonds with a conversion rate of 2100 μmol g_cat_^−1^ h^−1^ (Figure 3 a), suggesting Bi and Br atoms dominantly contribute to the catalytic reaction. BiBr_3_/SBA‐15 also displayed interactions with toluene (Supporting Information, Figure S4 d), but its poor activity could be related to its wider band gap (E_g_ of 3.1 eV; Supporting Information, Figure S7). Other common Bi‐based photocatalysts BiOBr and Bi_2_WO_6_ showed negligible activities (<100 μmol g_cat_^−1^ h^−1^), which agrees with the previous reports.7e, 7f Among all the reported active catalysts (even including thermal systems with harsh reaction conditions), our supported Cs_3_Bi_2_Br_9_/SBA‐15 photocatalyst demonstrates the highest reported catalytic activity (Supporting Information, Figure S8).

Halide perovskite‐based materials are known for their thermal and moisture instabilities, which are the main drawbacks for their use in wide‐range applications. As seen in Figure 3 b, the high benzaldehyde production rate was maintained up to 4 h of irradiation with visible light and the catalyst showed negligible structural changes after reaction (see XRD and UV/Vis DRS in the Supporting Information, Figure S9, and element analysis of leached species in the Supporting Information, Table S2). Furthermore, from the action spectrum with monochromatic light irradiation (Supporting Information, Figure S10), the amount of benzaldehyde varied in the same trend with the light adsorption profile, suggesting light energy as the driving force for C−H bond activation. Notably, a QE of 11 % was achieved under 1 sun irradiation by using a solar simulator. However, the production rate clearly slowed down after another 4 h of light illumination and the reaction stopped when the irradiation time was extended to 12 h. After the reaction, the yellow photocatalyst had turned to white in color as BiOBr was formed (Supporting Information, Figure S11) because of water generation. The deactivation of the photocatalysts can be prevented by addition of anhydrous Na_2_SO_4_ to absorb the produced water (Figure 3 b).

The application scope of supported perovskite nanoparticle photocatalysts was extended to other hydrocarbons from C_5_ to C_16_ including aliphatic and aromatic alkanes. As shown in Table 1 and the Supporting Information, Tables S3, S4, in all cases their corresponding oxygenates (aldehydes/ketones and alcohols) were formed with excellent selectivities (>99 %) without formation of any over‐oxidation products like CO_2_. Even though very low yields of oxygenates (<0.3 %; Supporting Information, Table S3) were obtained owing to the limited reaction time and very low catalyst concentration, the benzaldehyde yield can be enhanced to 3.8 % with modified reaction conditions like longer reaction time, higher catalyst concentration and higher light irradiation intensity (Supporting Information, Figure S12).

**Table 1:** Photo‐oxidation of hydrocarbons over supported Cs_3_Bi_2_Br_9_/SBA‐15 photocatalyst under visible‐light irradiation in air.^[a]^

Entry	Hydrocarbon	Conversion rate [μmol g_cat_^−1^ h^−1^]^[b]^	Oxygenated product distribution [%]^[c]^	
			aldehyde/ketone^[d]^	alcohol^[e]^
1	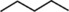	1500	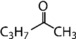	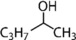
			67	33
2	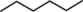	1600	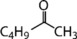	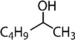
			74	26
3	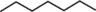	2100	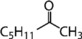	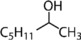
			74	26
4	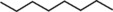	2300	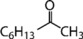	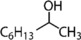
			76	24
5^[f]^	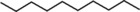	800	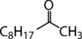	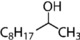
			>99	–
6^[f]^	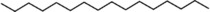	1100	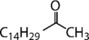	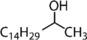
			>99	–
7	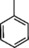	12 600	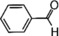	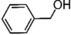
			90	10
8	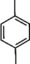	21 300	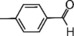	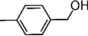
			85	15
9	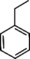	32 900	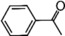	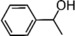
			>99	–
10	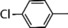	11 900	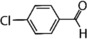	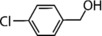
			80	20
11^[f]^	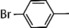	30 300	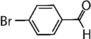	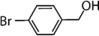
			85	15
12^[f]^	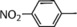	13 400	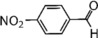	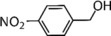
			89	11

[a] Reaction conditions: 5 mL liquid hydrocarbon, 10 mg 10 wt % Cs_3_Bi_2_Br_9_/SBA‐15 as photocatalyst, 1 bar air at 293 K under visible light (≥420 nm) irradiation. [b] Based on effective catalyst mass and total amount of produced ketones/aldehydes/alcohols determined by GC/MS and approximated to hundreds. [c] Selectivity for aldehydes/ketones and alcohols, respectively. [d] Using main ketone product as a representative (detailed product distribution in the Supporting Information, Table S4). [e] Using main alcohol product as a representative (detailed product distribution in Supporting Information, Table S4). [f] Reaction temperature of 373 K owing to higher melting points of substrates.

The aromatic substrates presented much higher conversion rate (Table 1, entries 7–12, >10 000 μmol g_cat_^−1^ h^−1^) than the aliphatic ones (Table 1, entries 1–6, <2500 μmol g_cat_^−1^ h^−1^), which generally have more robust C−H bonds with higher bond dissociation energies (BDEs; Supporting Information, Table S5).^27^ For the toluene derivatives, the substrate with an electron‐donating substituent (for example, −CH_3_) showed a higher reaction rate than the one with an electron‐withdrawing substituent (for example, −NO_2_), even though both have similar BDEs (Supporting Information, Table S5). However, the oxidation activities positively correlate with interactions between aromatics and perovskite nanoparticles, which could be indicated by UV/Vis DRS analysis (Supporting Information, Figure S4 f). Notably, for the linear aliphatic alkanes (from C_5_ to C_8_), the reaction rate increased as the carbon chain length increased, which could be ascribed to the more stable alkyl radical intermediates. For the longer alkanes (C_10_ and C_16_), the dramatically decreased activity might be originated from the diffusion limitations and steric hindrances owing to larger size and higher viscosity of substrates. Besides, linear ketones appeared as the dominant products with the secondary C−H bonds cleaved, which generally have lower BDEs than the terminal ones.^28^

### Interaction of Catalysts with Hydrocarbons

**Figure 4:**
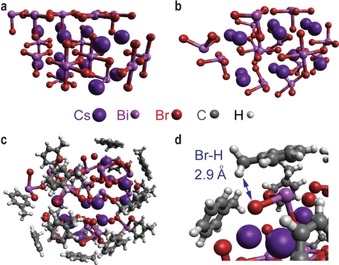
Electronic structure calculations. a) Cs_12_Bi_14_Br_54_ cluster from crystal structure of Cs_3_Bi_2_Br_9_. b) Optimized Cs_12_Bi_14_Br_54_ cluster at the PBE‐D3/def2‐svp level. c) Optimized geometry of the Cs_12_Bi_14_Br_54_ cluster with 17 toluene molecules. d) Focus on a typical Br−H geometry.

A DFT study was conducted to investigate the electronic structure of halide perovskite and its interactions with hydrocarbons (Supporting Information, Figures S13–S17 and Tables S6, S7). Figure 4 shows modeling of a stable Cs_12_Bi_14_Br_54_ cluster with BiBr_3_ and BiBr_5_ motifs on the surface (size ca. 1.5 nm; Figure 4 b), and optimized geometries for toluene adsorption on the nanoparticle (Figure 4 c,d). Specifically, this model, with a high surface to bulk ratio, is mainly designed to simulate the surface environment of Cs_3_Bi_2_Br_9_ nanoparticles with a wide variety of motifs. Furthermore, all adsorbed toluene molecules exhibit an interaction pattern with their benzene rings either on top of Bi or Cs atoms (average distance of 3.0 and 3.5 Å, respectively) (Figure 4 c), resulting in Bi‐arene (Cs‐arene) interacting through dispersion interactions and charge transfer (from the π system to Bi σ* orbitals).^29^ Notably, hydrogen atoms of −CH_3_ groups are located in the vicinity of surface Br atoms (distance around 2.9 Å; Figure 4 d). Our calculations also indicate that the presence of toluene molecules at the surface induces low‐energy absorption peaks (from 500 nm to 650 nm) as shown in the Supporting Information, Figure S16. The analysis of computational results (Supporting Information, Figures S13–S17 and Tables S6–S7) shows that low‐energy transitions mostly correspond to the charge transfer from lone electron pairs of a Br atom with a small contribution of the π system of toluene to an empty orbital of a Bi atom. An additional work needs to be conducted to investigate the interactions between halide perovskites and aliphatic hydrocarbons, which will be scope of another study.

### Reaction Mechanism

**Figure 5:**
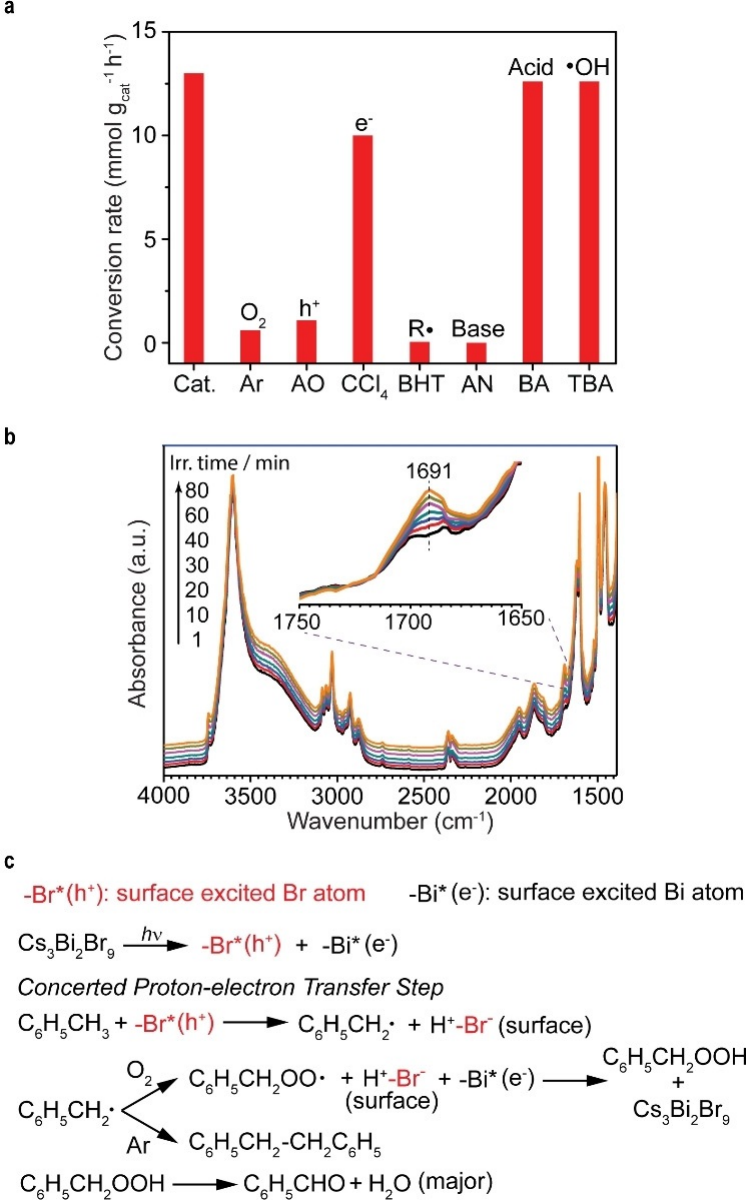
Proposed reaction mechanism. a) Control tests in the anaerobic condition or with different scavengers such as ammonium oxalate (AO), CCl_4_, butylated hydroxytoluene (BHT), aniline (AN), benzoic acid (BA) and *tert*‐butanol (TBA). b) In situ DRIFTs spectra of Cs_3_Bi_2_Br_9_/SBA‐15 sample in the gas mixture of O_2_ and toluene under light irradiation (Inset: Feature in the range from 1750 to 1650 cm^−1^). c) Possible reaction pathways of toluene oxidation based on the experimental results.

To further investigate the photocatalytic process, a series of control experiments have been conducted in the absence of O_2_ or with addition of various scavengers, which demonstrated different effects on the conversion rate of toluene oxidation as shown in Figure 5 a. Almost no benzaldehyde and benzyl alcohol production happened under an Ar atmosphere, directly proving the necessity of O_2_ for oxygenated compounds formation. However, in anaerobic conditions the coupling reaction occurred with formation of 1,2‐diphenylethane and change in photocatalyst color from yellow to black (Supporting Information, Figure S18).

Moreover, under Ar atmosphere the addition of 2,2,6,6‐tetermethylpiperidine‐*N*‐oxyl (TEMPO) as a radical trapper captures the benzyl radical with the corresponding adduct formed (Supporting Information, Figure S19 a). Meanwhile, 1‐hydroxy‐2,2,6,6‐tetramethyl‐piperidine (TEMPO‐H; Supporting Information, Figure S19 b) was also generated owing to the reaction with reducing species (H^+^/e^−^) and the photocatalyst color was maintained.^30^ These results verify formation of carbon‐centered radicals as intermediates after the cleavage step of C(sp^3^)−H bonds. The abstracted H atoms may be transferred to the perovskite surface (see the Supporting Information, solid‐state ^1^H nuclear magnetic resonance spectroscopy in Figure S20 and in situ MS in Figure S21), probably leading to the color change of the photocatalyst after irradiation in Ar.2b Besides, no reduced Bi species (such as Bi^2+^) were observed by the X‐ray photoelectron spectroscopy (XPS) analysis on the excited photocatalyst (cleaned and dried under vacuum; Supporting Information, Figure S22 a,b). The addition of ammonium oxalate (NH_4_)_2_C_2_O_4_ as hole scavenger and butylated hydroxytoluene (BHT) as carbon‐centered radical scavenger both terminated the reaction, while CCl_4_ as electron acceptor slightly affected the activity. Clearly, photoinduced holes (h^+^) are significant to activate C(sp^3^)−H bonds in toluene.

An isotopic labeling experiment was conducted in order to gain more insights into the reaction mechanism. When deuterated toluene was used as reactant, an apparent kinetic isotope effect (KIE) was observed with a *k*_H_/*k*_D_ value of 4.4 (Supporting Information, Table S8), suggesting C(sp^3^)−H bond activation as the rate‐determining step. In contrast, the further reaction of benzyl radicals with O_2_ or superoxide anions ^.^O_2_^−^ (from reduction of O_2_ by photoinduced electrons) took place very rapidly, coinciding with the previous report.^31^ Besides, hydroxyl radicals had no contribution, as *tert*‐butanol addition did not suppress activity.

Notably, the addition of aniline as a base prohibited the reaction completely, while no influence appeared in the case of benzoic acid. This could be explained by the poisoning of Bi atoms serving as weak Lewis acid sites.^20^ The peroxy species (ROO^.^) can be generated from the fast reaction of alkyl radicals with O_2_.^31^ In our perovskite system, ROO^.^ react with reducing species (H^+^/e^−^) on perovskite surface to form ROOH, which can be further decomposed to carbonyl products (Figure 5 c).^32^ This dominant reaction path was supported by in situ diffuse reflectance infrared Fourier transform spectroscopy (DRIFTs) experiments as shown in Figure 5 b. In the presence of toluene and O_2_ gas mixture, one peak at 1691 cm^−1^ corresponding to the carbonyl group of benzaldehyde appeared after the light irradiation of Cs_3_Bi_2_Br_9_/SBA‐15 powders, while no peaks assigned to benzyl alcohol could be detected in the whole irradiation process. Moreover, the energy barrier for C−H bond activation step on Cs_3_Bi_2_Br_9_ nanoparticle can be regarded as the energy difference between reactants (toluene on excited surface −Br*[h^+^]) and intermediates (PhCH_2_^.^ with −Br^−^−H^+^ surface). Clearly, this C−H bond activation induced by holes (on Br atoms), which is analogous to NO_3_^.^ radical‐driven oxidation as a concerted proton‐electron transfer process,^33^ is thermodynamically feasible as indicated by our calculations (see calculated potential energy of possible intermediates in the Supporting Information, Figure S23).

**Figure 6:**
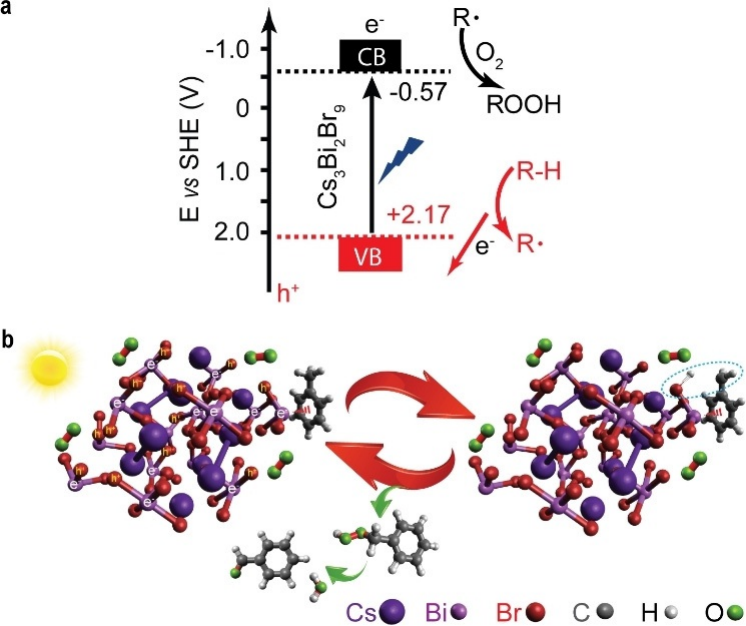
Energy diagram and proposed mechanism of supported Cs_3_Bi_2_Br_9_ nanoparticle for C(sp^3^)−H bond activation. a) Band positions of Cs_3_Bi_2_Br_9_ nanoparticle including the proposed redox reaction paths based on concerted proton–electron transfer process. b) Possible catalytic mechanism of Cs_3_Bi_2_Br_9_ nanoparticle as photocatalyst for hydrocarbon oxidation under visible light irradiation in air. The blue dotted circle indicates the production of an intermediate benzyl radical after cleaving C−H bond.

Based on the above experimental data and electronic structure calculations, we can put forward a hypothesis for the reaction mechanism for hydrocarbon oxidation using supported halide perovskite nanoparticles as photocatalysts. As shown in Figure 6 a, under visible light irradiation of Cs_3_Bi_2_Br_9_ nanoparticles, the photoinduced electrons and holes are generated in the conduction band minimum (CBM, −0.57 eV vs. SHE) and valence band maximum (VBM, +2.17 eV vs. SHE) positions, respectively (Supporting Information, Figure S22 c,d). The Bi p orbitals dominantly contribute to the CBM, while Br 4p orbitals correspond to VBM.8e, 34 Subsequently, with promoted electron–hole separation on Cs_3_Bi_2_Br_9_ nanoparticles, the electrons reduce the electron acceptors (for example, O_2_ and H^+^) and the holes drive the challenging oxidation of C(sp^3^)−H bonds to form alkyl radical intermediates (R^.^). As illustrated in Figure 6 b (with toluene as a model), before light illumination, the preferential geometry of toluene adsorption results in the proximity between H atoms of the −CH_3_ group and Br atoms, which simultaneously serve as oxidation points on the perovskite. Finally, the close contact between reactive oxidation sites (Br atoms) and H atoms from hydrocarbons promotes the activation of C(sp^3^)−H bonds.

## Conclusion

A series of photocatalysts consisting of lead‐free Cs_3_Bi_2_Br_9_ nanoparticles confined in a matrix of mesoporous SBA‐15 silica have been prepared. The well‐dispersed halide perovskite nanoparticles (2–5 nm) lead to better charge separation, more accessible active sites, and close contact with hydrocarbons, which facilitate the activation of C−H bonds in hydrocarbons. Under visible light irradiation, the supported Cs_3_Bi_2_Br_9_/SBA‐15 photocatalysts efficiently oxidize C(sp^3^)−H bonds of various hydrocarbons (from C_5_ to C_16_ including aromatic and aliphatic alkanes) to their corresponding oxygenates (mainly aldehydes/ketones) with a conversion rate of up to 32 900 μmol g_cat_^−1^ h^−1^ and high selectivity of >99 %. We believe this work could provide a promising direction for exploring halide perovskite photocatalysis in more challenging organic transformation reactions.

## Conflict of interest

The authors declare no conflict of interest.

## Supporting information

As a service to our authors and readers, this journal provides supporting information supplied by the authors. Such materials are peer reviewed and may be re‐organized for online delivery, but are not copy‐edited or typeset. Technical support issues arising from supporting information (other than missing files) should be addressed to the authors.

Supplementary
