# Supplementary material for: A Supported Bismuth Halide Perovskite Photocatalyst for Selective Aliphatic and Aromatic C–H Bond Activation
Source: Angew Chem Int Ed Engl. 2020 Jan 23;59(14):5788–96. doi: 10.1002/anie.201915034 (PMC7154683; doi:10.1002/anie.201915034)
Supplement: Supplementary file 1 — Supplementary [file ANIE-59-5788-s001.pdf]

Angewandte Chemie
Eine Zeitschrift der Gesellschaft Deutscher Chemiker

Supporting Information

A Supported Bismuth Halide Perovskite Photocatalyst for Selective Aliphatic and Aromatic C–H Bond Activation

*Yitao Dai, Corentin Poidevin, Cristina Ochoa-Hernández, Alexander A. Auer, and Harun Tüysüz**

anie_201915034_sm_miscellaneous_information.pdf

1. Photocatalysts Preparation

Raw Materials: the following commercial reagents were employed from Sigma-Aldrich: $Bi(NO_3)_3{\cdot}5H_2O$ (ACS reagent, ≥98.0%), $Na_2WO_4{\cdot}2H_2O$ (ACS reagent, ≥99%), $BiBr_3$ (purity ≥98%), CsBr (99.9% trace metals basis), $PbBr_2$ ((purity ≥98%), $AgNO_3$ (ACS reagent, ≥99.0%), KBr (BioXtra, ≥99.0%), DMSO (purity ≥99%), DMF (anhydrous, 99.8%), isopropanol (ACS reagent, ≥99.5%), NH_4OH (ACS reagent, 28.0-30.0% NH_3 basis), Pluronic 123 (P123, poly(ethylene glycol)-poly(propylene glycol)-poly(ethylene glycol); EO:PO:EO = 20:70:20, average M_n = ~5800), Tetraethyl orthosilicate (TEOS, 98%)

Supported halide perovskite catalysts: Mesoporous SBA-15 was synthesized based on the literature[1]. $Cs_3Bi_2Br_9$/SBA-15 samples were prepared using the common incipient wetness impregnation method[2]. To prepare the supported samples with different loadings of $Cs_3Bi_2Br_9$, corresponding amounts of CsBr and $BiBr_3$ (molar ratio of 3:2) dissolved in DMSO was added into SBA-15 powder in a mortar and pressed with a pestle. More specifically, 250 mg dried SBA-15 was mixed with 0.33 mL DMSO solution, containing CsBr and $BiBr_3$ precursors (for example, 10 wt.% $Cs_3Bi_2Br_9$/SBA-15 sample needs addition of 12.0 mg CsBr and 16.8 mg $BiBr_3$ in DMSO). After 20 min physical mixing in the mortar, the mixture was dried in an oven at 150 ^oC for 12 h. The dried clean photocatalysts were used for the materials characterization and photocatalytic reaction tests. For the cases of supported CsBr/SBA-15, $BiBr_3$/SBA-15, $CsPbBr_3$/SBA-15 and $Cs_2AgBiBr_6$/SBA-15, the same preparation procedure was employed except by using different dissolved metal halide precursors.

Bulk $Cs_3Bi_2Br_9$: $Cs_3Bi_2Br_9$ crystals were prepared *via* the anti-solvent method[3]. 10 mL solution of DMSO containing dissolved CsBr (488 μmol) and $BiBr_3$ (325 μmol) was dropwise added into 50 mL isopropanol under stirring. The formed yellow suspension was filtrated, washed by isopropanol and dried at 80 ^oC in air for 12 h to obtain bulk $Cs_3Bi_2Br_9$ crystals.

Bi_2WO_6: it was prepared according to the literature[4], 1 mmol (0.49 g) $Bi(NO_3)_3{\cdot}5H_2O$ was dissolved in 15 mL of 2 $mol{\cdot}L^{-1}$ HNO_3 solution. Subsequently, a solution of 0.5 mmol $Na_2WO_4{\cdot}2H_2O$ in 15 mL deionized (DI) water was added into the above solution under stirring. After 24 h mixing, the formed white suspension was transferred to a 50 mL Teflon-lined autoclave and maintained at 160 ^oC for 12 h. The final powder was obtained by filtration, cleaning with DI water and drying at 80 ^oC in air for another 12 h.

BiOBr[5]: 5 mmol $Bi(NO_3)_3{\cdot}5H_2O$ was added into a 100 mL solution with NaBr in a same molar amount. Then 1 $mol{\cdot}L^{-1}$ NH_4OH solution was added to neutralize the above mixture. After vigorous stirring for 24 h, the white suspension was collected by filtration, washing with DI water and drying at 80 ^oC for 12 h.

2. Physical and Chemical Properties Characterisation Methods

2.1 Powder X-ray diffraction (XRD) and electron microscopy

To study the crystal structure and phase composition of photocatalyst samples, XRD data (Figure S1) were collected using a STOE θ/θ diffractometer equipped with a Cu Kα source. The XRD patterns were recorded using the typical θ-2θ geometry (Bragg-Brentano). The small-angle XRD analysis employed a scan range from 0.6 to 10° with the step size of 0.004^o and scan rate of $0.002^o{\cdot}s^{-1}$, while wide-angle XRD scans from 5 to 90° with the step size of 0.02^o and scan rate of $0.01^o{\cdot}s^{-1}$. Scanning transmission electron microscopy (STEM) and energy dispersive X-ray spectrometry (EDS) mappings were recorded on a Hitachi HD-2700 C_s-corrected STEM operated at 200 kV. The SEM-EDX analyses were taken on a Hitachi electron microscope S3500N. The EDX analyses were performed with a Si (Li) Pentafet Plus detector from Oxford Instruments GmbH. The standard measuring conditions of 25kV excitation voltage, 600s measuring time and 100x magnification were used.

2.2 N_2-physisorption

The Brunauer-Emmett-Teller (BET) surface areas of $Cs_3Bi_2Br_9$/SBA-15 samples with different loadings were determined from nitrogen adsorption/desorption isotherms measured at 77 K using a 3Flex Micromeritics system (Figure S2).

2.3 Alizarin adsorption

20 mg photocatalyst sample was added into 5 mL isopropanol with 1 mg alizarin (dye reagent) dissolved. After vigorous stirring for half hour, the solid sample was isolated by centrifuging and washing with isopropanol to remove the excessive alizarin. Then the sample adsorbed with alizarin was dried at 80 ^oC for 12 h, which was subsequently measured by UV-Vis diffuse reflectance spectroscopy (DRS). The light absorption profiles for the alizarin-isopropanol solution and clean photocatalyst powders were analysed by UV-Vis spectroscopy in a transmission mode and DRS mode, respectively. Then, the absorption spectrum corresponding to the clean photocatalyst sample was subtracted from the one recorded with alizarin adsorption to obtain the final plot (Figure S3), avoiding the overlap due to the possible light absorption from the sample itself.

2.4 UV-Vis DRS and PL

The DRS profiles were recorded at room temperature (R.T.) with a wavelength range of 300-800 nm using a PerkinElmer Lambda 365 UV-vis spectrophotometer with $BaSO_4$ (spectroscopy grade) as the reference. For each measurement, 100 mg sample was loaded for analysis. According to the Kubelka-Munk theory[6], Tauc plots of a direct semiconductor can be drawn by plotting $[F(R)\cdot hv]^2$ versus *hv* (incident photo energy)[7], where $F(R) = (1-R)^2/(2R)$ and *R* is the measured reflectance. To investigate the possible interaction between hydrocarbons and supported photocatalyst samples, a drop (0.15 mL) of a hydrocarbon liquid was directly mixed with dry sample powders to get a wet sample with saturated adsorption of hydrocarbons, which was subsequently analysed by UV-Vis DRS. The results for the cases of different photocatalyst samples with hydrocarbon adsorption are shown in Figure S4b-d. For 10 wt.% $Cs_3Bi_2Br_9$/SBA-15 samples adsorbing F-substituted hydrocarbons (trifluorotoluene, perfluorobenzene and perfluorohexane) or *para*-substituted benzene substrates (aniline, toluene, nitrobenzene and trifluorotoluene), UV-Vis DRS results are presented in Figure S4e-f. PL spectra were obtained on an Edinburgh FS5 spectrofluorometer with 2.5 mg sample dispersed in 10 mL isopropanol.

2.5 Fourier Transform Infrared Spectroscopy (FT-IR)

2.5.1. *In-situ* Diffuse Reflectance Infrared Fourier Transform Spectroscopy (DRIFTs) analysis

To study the possible reaction pathway during the photo-oxidation of toluene, *in-situ* DRIFTs test was conducted using the Praying Mantis accessory (Harrick) in a Nicolet Magna-IR 560 spectrometer equipped with an MCT detector. The fresh powder sample (10 wt.% $Cs_3Bi_2Br_9$/SBA-15) was placed in the catalytic reaction chamber equipped with two KBr windows and one SiO_2 window dome and coupled with a temperature controller for heating. Firstly, the sample was heated to 200 °C for 2 h under N_2 flow in order to remove the adsorbed water molecules. After the sample was cooled down to R.T., toluene vapor with O_2 was introduced into the chamber *via* a home-made bubbling system, in which O_2 flow bubbled through toluene liquid at R.T. After a sufficient introduction of toluene and O_2 for 3 h, the sample was irradiated for 80 min and IR spectra were recorded simultaneously and repeatedly with a certain time interval (Figure 5b in main text).

2.5.2. Transmission Infrared Spectroscopy analysis

Self-supported wafers of 10 wt.% $Cs_3Bi_2Br_9$/SBA-15 and SBA-15 (8-12 mg/m^2) were prepared and activated under high vacuum at 200 °C for 2 h to remove the physiosorbed water. Subsequently, small amounts of toluene were dosed gradually into the system at room temperature. Spectra were recorded by using a Nicolet iS50 equipped with an MCT detector.

2.6 X-ray Photoelectron Spectroscopy (XPS)

XPS measurements were performed with a spectrometer from SPECS GmbH equipped with a PHOIBOS 150 1D-DLD hemispherical energy analyser. The monochromatized Al Kα X-ray source (E=1486.6 eV) was operated at 15 kV and 200W. For the narrow scans, 20 eV pass energy was applied. The medium area mode was used as lens mode. The base pressure during the experiment in the analysis chamber was 5×10^{-10} mbar. To account charging effects, all spectra have been referred to C 1s at 284.5 eV (Figure S22a-c).

2.7 Solid-state ^{1}H NMR and element analysis

The solid-state ^{1}H NMR spectra were recorded on a Bruker Avance 500WB spectrometer with magic-angle spinning (MAS) probeheads. Elemental analysis of the leaching species in reaction solution was performed by the Microanalytical Laboratory Kolbe (Nachf.) in Germany by using atomic absorption spectroscopy (AAS Model AAnalyst 200 from Perkin Elmer) for Bi and Cs elements and ion chromatography (Metrohm Model 883 Plus) for Br element.

3. Photocatalytic Evaluation Methods

The photocatalyst was added to 5 mL of pure hydrocarbon (*e.g.*, toluene) to form yellow suspension in a steel autoclave (25 mL), which was sealed by a borosilicate window for on-top illumination. Subsequently, the autoclave with reaction suspension was placed in a cooling jacket (293 K) and excited with a 300 W Xe-lamp equipped with a 420 nm cut-off filter (Newport 20CGA-420) in 1 bar air atmosphere (Figure S5). Monochromatic light with the wavelength of 420 nm, 450 nm, 475 nm, 500 nm, 550 nm, 600 nm, 650 nm and 700 nm for the action spectra was emitted from the same lamp coupling with a Newport CornerStone 130 monochromator. After irradiation, the liquid phase was quantitatively analyzed by gas chromatography (GC) or gas chromatography-mass spectrometry (GC-MS) with trifluorotoluene as internal standard. The gas phase was analyzed by GC7820A (two connected columns: a capillary column HP-PLOT Q and a packed column Molsieve 5Å) with a gas sampling system. Meanwhile, a mobile MS setup (ThermoStar from Balzers Instruments) was employed additionally for gas analysis. An example of the gas phase analysis is shown in Figure S6a-b. The toluene conversion rate was calculated based on the following equation using weight of halide perovskite phase as the effective catalyst mass, because the support SBA-15 is totally inert without light absorption. For example, for the case of 10 wt.% $Cs_3Bi_2Br_9$/SBA-15 sample (10 mg photocatalyst with 1 mg $Cs_3Bi_2Br_9$ loaded), after 2h irradiation the converted toluene was 25.2 µmol, which means a conversion rate of 12600 µmol g_{cat}^{-1} h^{-1} by considering 1mg $Cs_3Bi_2Br_9$ as the effective catalyst mass.

$$\text{Conversion rate } (\mu mol\ g_{cat.}^{-1} h^{-1}) = \frac{n_{con.\ tol.}}{m_{cat.} * t_R}$$

where $n_{con.tol.}$ is the converted number of toluene (μmol), m_{cat} is the effective catalyst mass (mg, specifically weight of loaded halide perovskite for the supported catalysts), t_R is the irradiation time (h).

For the test under 1 sun irradiation, the light source was changed to a solar simulator (LCS-100, Newport). The light intensity was calibrated to 1 sun using the 91150V Reference Cell and Meter from Newport, consisting of a readout device and a 2 x 2 cm calibrated solar cell with a monocrystalline silicon and a fused silica window. The reaction conditions were similar with the typical evaluation test except using sufficient catalyst sample (100 mg) for maximum light absorption. Due to the absorption ability of the $Cs_3Bi_2Br_9$/SBA-15 catalyst, only part fractions of sunlight (λ ≤ 480 nm) were considered to determine the quantum efficiency (QE) as follows. Base on the literature[8], the part of a solar spectrum from UV to blue light (from 280 to 480 nm) can be divided into three intervals: i = 1 is the zone from 280 to 400 nm with 340 nm as the average wavelength (λ_i=1); i = 2 means from 400 to 440 nm with λ_i=2 at 420 nm; and i = 3 designates from 440 to 480 nm with 460 nm as λ_i=3. The effective total incident photon number per hour ($N_{1\,sun}$) was calculated according to this equation:

$$N_{1\,sun} = \sum_i \frac{E_{sun} * p_i * S_R * \lambda_i * t}{h * c} = \sum_i \frac{0.1 * p_i * 1.5 * \lambda_i * 3600}{6.626 * 10^{-34} * 3 * 10^{8}}$$

where E_{sun} is the averaged sunlight intensity (0.1 W·cm^2), p_i is the fraction of solar power in the intervals i = 1-3 (p_1=7.63%, p_2=5.08%, p_3=5.85%)[8], λ_i is the averaged wavelength of interval i and S_R is the irradiation area (1.5 cm^2).

The estimated $N_{1\,sun}$ is 2.1*10^{20} h^{-1}. Therefore, in the case of toluene oxidation under 1 sun irradiation, the QE was estimated to be 11% based on the following equation:

$$\mathrm{QE} = \frac{n_{con.\ tol.} * n_{e,i}}{N_{1\,sun} * t_R} * 100\%$$

where $n_{con.\ tol.}$ is the converted number of toluene (150 μmol), $n_{e,i}$ is the number of electrons needed for converting one toluene to the corresponding product (benzaldehyde or benzyl alcohol), t_R is the reaction time (2 h).

To study the influence of irradiation wavelength, as shown in Figure S10a the action spectrum was obtained by using monochromatic light with the respective wavelength of 420 nm, 450 nm, 475 nm, 500 nm, 550 nm, 600 nm, 650 nm and 700 nm from the same 300 W Xe-lamp attached to a Newport CornerStone 130 monochromator. The light intensity was adjusted to a similar level for comparison (~10 mW·cm^{-2}, Figure S10b).

4. Electronic structure calculations – computational details

The calculations were carried out using the ORCA 4.0 program package[9]. The geometry optimizations were performed at the PBE-D3/def2-SVP//def2-SD level[10]. Subsequently, single point and TD-DFT calculations were performed at the B3LYP-D3/def2-SVP//def2-SD level using RIJCOSX approximation and C-PCM model of toluene[11]. The detailed calculation results are shown in Figure S13-17.

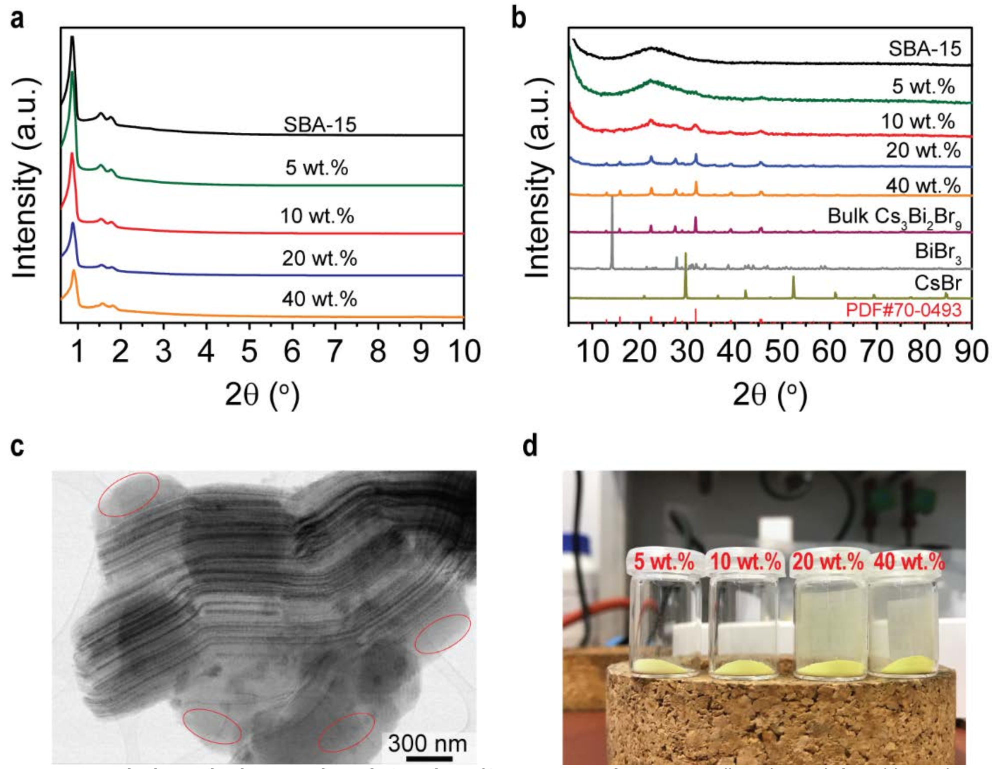

Figure S1. XRD, TEM analysis and photographs of $Cs_3Bi_2Br_9$/SBA-15 samples. a, Small-angle and **b,** wide-angle XRD patterns of $Cs_3Bi_2Br_9$/SBA-15 samples (5 wt.%, 10 wt.%, 20 wt.% and 40 wt.% loadings) with bare SBA-15, bulk $Cs_3Bi_2Br_9$, $BiBr_3$ and CsBr as references. **c,** TEM image of the 40 wt.% $Cs_3Bi_2Br_9$/SBA-15 (black lines indicate the perovskites confined in the pore channels and red circles indicate the empty unfilled SBA-15 domains). **d,** Photographs of the samples with different loadings.

In comparison with the bare silica support and bulk $Cs_3Bi_2Br_9$ (aggregated large particles 50-500 nm), XRD patterns (Figure S1b) of all supported samples solidly confirm the presence of silica and halide perovskite phase with a trigonal *P3m1* symmetry (PDF#70-0493 card)[12]. Besides, the presence of precursors can be ruled out in comparison with XRD profiles of pure $BiBr_3$ and CsBr. The increase of loading to 20 wt.% and 40 wt.% results in much sharper and more apparent diffraction of perovskite phase, suggesting the appearance of larger perovskite particles due to external pore growth without confinement or serious aggregation in pore channels. Furthermore, small-angle XRD patterns in Figure S1a indicate that the ordered mesoporous structure of SBA-15 support is well preserved in all supported samples. This could also be corroborated by nitrogen adsorption-desorption isotherms and pore size distributions (PSD) as shown in Figure S2, in which all $Cs_3Bi_2Br_9$/SBA-15 samples exhibit typical IV isotherms with a hysteresis loop of type H1 and the same PSD focusing at ~7.9 nm[13]. Moreover, as indicated by Figure S1c, the as-prepared samples contain the unfilled SBA-15 domains due to the physical mixing process.

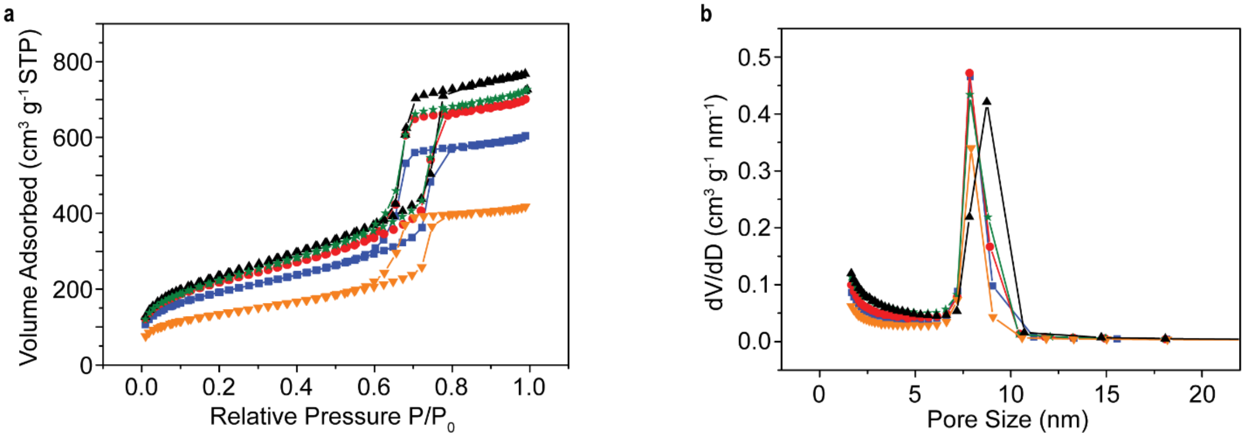

Figure S2. N_2-physisorption analysis of $Cs_3Bi_2Br_9$/SBA-15 samples. a, N_2 adsorption-desorption isotherms and **b,** PSD of $Cs_3Bi_2Br_9$/SBA-15 samples with the loadings of 5 wt.% (-★-), 10 wt.% (-●-), 20 wt.% (-■-) and 40 wt.% (-▼-), including SBA-15 (-▲-) as the reference.

Table S1. Element analysis of supported $Cs_3Bi_2Br_9$/SBA-15 samples with different loadings by use of SEM-EDX characterizations.

Calculated loading of $Cs_3Bi_2Br_9$ (wt.%)	Cs (wt.%)	Bi (wt.%)	Br (wt.%)	Total Cs + Bi + Br (wt.%)	Atomic ratio of Cs / Bi / Br
**5**	1.60	1.75	2.38	5.74	3.0 / 2.2 / 7.6
**10**	2.51	2.71	3.92	9.14	3.0 / 2.0 / 7.8
**20**	4.87	5.14	8.50	18.51	3.0 / 2.0 / 8.7
**40**	8.73	8.34	17.15	34.22	3.0 / 1.8 / 9.8

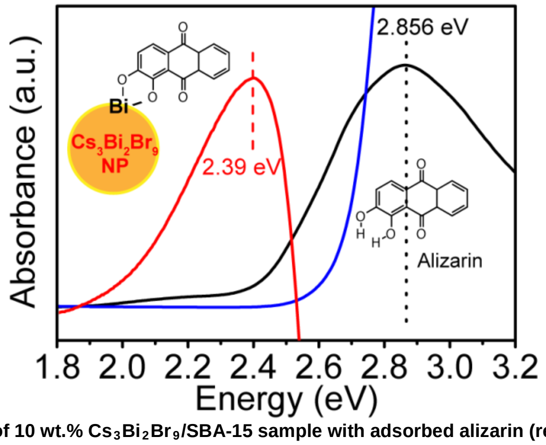

Figure S3. **UV-Vis absorption analysis of 10 wt.% $Cs_3Bi_2Br_9$/SBA-15 sample with adsorbed alizarin (red line), clean 10 wt.% $Cs_3Bi_2Br_9$/SBA-15 sample (blue line) and pure alizarin (black line).**

To investigate the surface acidity, UV-Vis DRS analysis was performed using alizarin as a probe molecule as reported in our previous study[14]. Pure alizarin shows absorbance at 2.856 eV, which is due to the intramolecular charge-transfer (IMCT) from the catechol moiety to the whole ring system. With alizarin adsorbed onto the $Cs_3Bi_2Br_9$/SBA-15 perovskite surface, the IMCT band is clearly shifted to 2.39 eV, indicating the existence of weak Lewis acid on surface (Figure S3)[14].

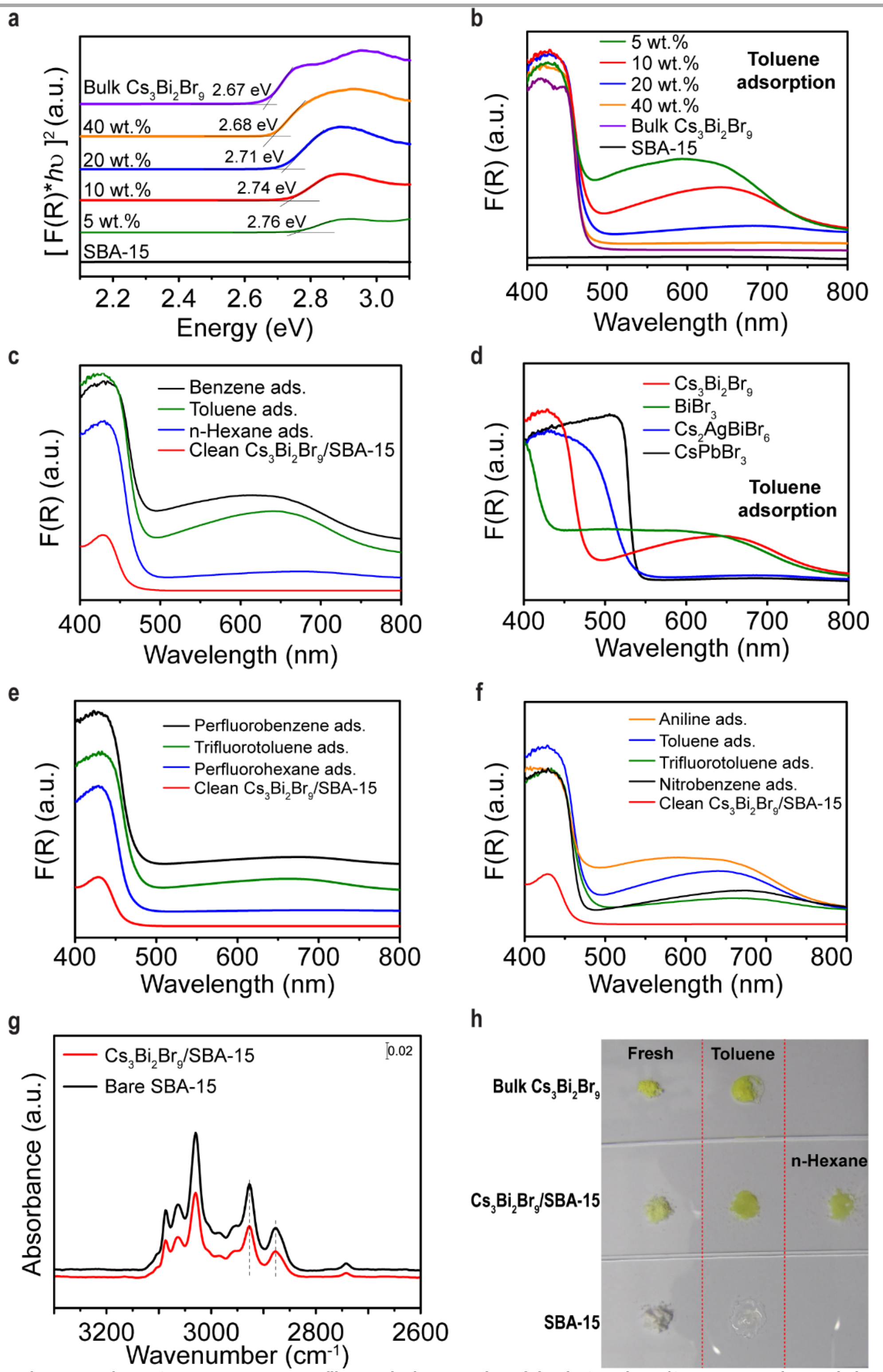

Figure S4. Tauc plots, UV-Vis DRS spectra, FT-IR profiles and photographs of fresh $Cs_3Bi_2Br_9$/SBA-15 samples and the samples with hydrocarbons adsorbed. a, Tauc plots of $Cs_3Bi_2Br_9$/SBA-15 with different loadings, bare SBA-15 and bulk $Cs_3Bi_2Br_9$. **b,** UV-Vis DRS spectra of $Cs_3Bi_2Br_9$/SBA-15 samples after toluene adsorption, including bare SBA-15. **c,** UV-Vis DRS spectra of 10 wt.% $Cs_3Bi_2Br_9$/SBA-15 with different hydrocarbons adsorbed on the surface. **d,** UV-Vis DRS spectra of various 10 wt.% supported halides ($Cs_3Bi_2Br_9$, $BiBr_3$, $CsPbBr_3$ and $Cs_2AgBiBr_6$ on SBA-15) with toluene adsorbed. **e,** UV-Vis DRS data of 10 wt.% $Cs_3Bi_2Br_9$/SBA-15 sample adsorbed with different F-substituted hydrocarbons and **f,** substituted benzene substrates with $-NH_2$, $-CH_3$, $-CF_3$ and $-NO_2$ groups. **g,** FT-IR spectra in the C-H vibration range of adsorbed toluene on the 10 wt.% supported sample and bare SBA-15 as reference. **h,** Images of the wetted supported samples (5 wt.% loading) with toluene and n-hexane in comparison with bare SBA-15 and bulk $Cs_3Bi_2Br_9$.

Toluene has been chosen as the probe substrate for C-H bond activation and we investigated its interaction with $Cs_3Bi_2Br_9$/SBA-15 composite through experimental spectroscopic methods and electronic structure calculations. The UV-Vis DRS spectroscopic study reveals that after adsorption of toluene, which only has UV absorption below 300 nm, the $Cs_3Bi_2Br_9$/SBA-15 composites with nanoparticle loadings from 5 to 20 wt.% display in an unexpected strong absorption band in the visible range (from 500 to 750 nm, Figure S4b). In contrast, 40 wt.% $Cs_3Bi_2Br_9$/SBA-15, bare SBA-15 and bulk $Cs_3Bi_2Br_9$ did not display this absorption band due to lack of well-dispersed perovskite nanoparticles. We assume this absorption may come from electronic interactions with charge transfer between the electron-rich benzene ring and under-coordinated Bi atoms serving as Lewis acid sites on the nanoparticle surfaces (Figure S3).[14] The FT-IR spectrum shows no shift of C-H bonds for toluene adsorbed on perovskite nanoparticles (Figure S4g), indicating no intramolecular interaction with C-H bonds[15].

Notably, when other hydrocarbons (*e.g.*, benzene and n-hexane) were used as probes, similar visible light absorption bands were observed (Figure S4c). The weaker light absorption in the case of n-hexane may be ascribed to the lower electron density of the carbon chain in aliphatic alkanes. It should be stated that we are still not clear about the origin of this unexpected visible light absorption for linear aliphatic cases (n-hexane). Further investigations are in progress. Besides, this electron density influence was well evidenced in the cases of F-substituted hydrocarbons and substituted benzene substrates (Figure S4e-f). Furthermore, as shown in Figure S4d, this visible light absorption band also clearly appeared in the case of the supported $BiBr_3$/SBA-15 sample, suggesting that this light absorption band mainly involves surface Bi and Br atoms. In contrast, SBA-15 silica supported $Cs_2AgBiBr_6$ and $CsPbBr_3$ nanoparticles exhibit a much weaker absorption band, which could be ascribed to partial Ag replacement of Bi and the weaker Lewis acidity of Pb atoms[14, 16]. Therefore, we can conclude that the small single nanoparticle of $Cs_3Bi_2Br_9$ (2-5 nm) may have a close contact and interaction with hydrocarbons, contributing to the visible light absorption band from 500 to 750 nm.

Regarding the electronic interaction between hydrocarbons and supported $Cs_3Bi_2Br_9$ nanoparticles, it can be observed that the partial or total substitution of H atoms in these hydrocarbons by F atoms (trifluorotoluene, perfluorobenzene and perfluorohexane) rendered the absorption band significantly weakened or vanished (Figure S4e). This could be ascribed to the decreased electron density of carbon framework with F atom bonded. Thus, this interaction has a positive relationship with the electron density of hydrocarbons, which was well corroborated by the case of substituted benzene substrates with electron-donating and electron-withdrawing groups (aniline, toluene, trifluorotoluene and nitrobenzene in Figure S4f).

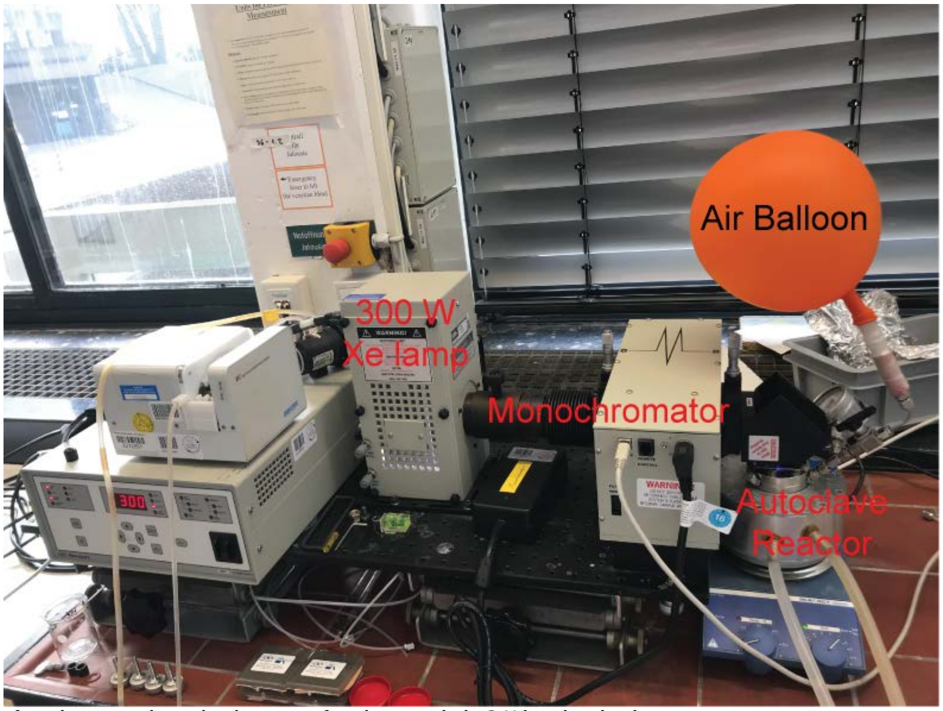

Figure S5. **Image of our home-made evaluation setup for photocatalytic C-H bond activation.**

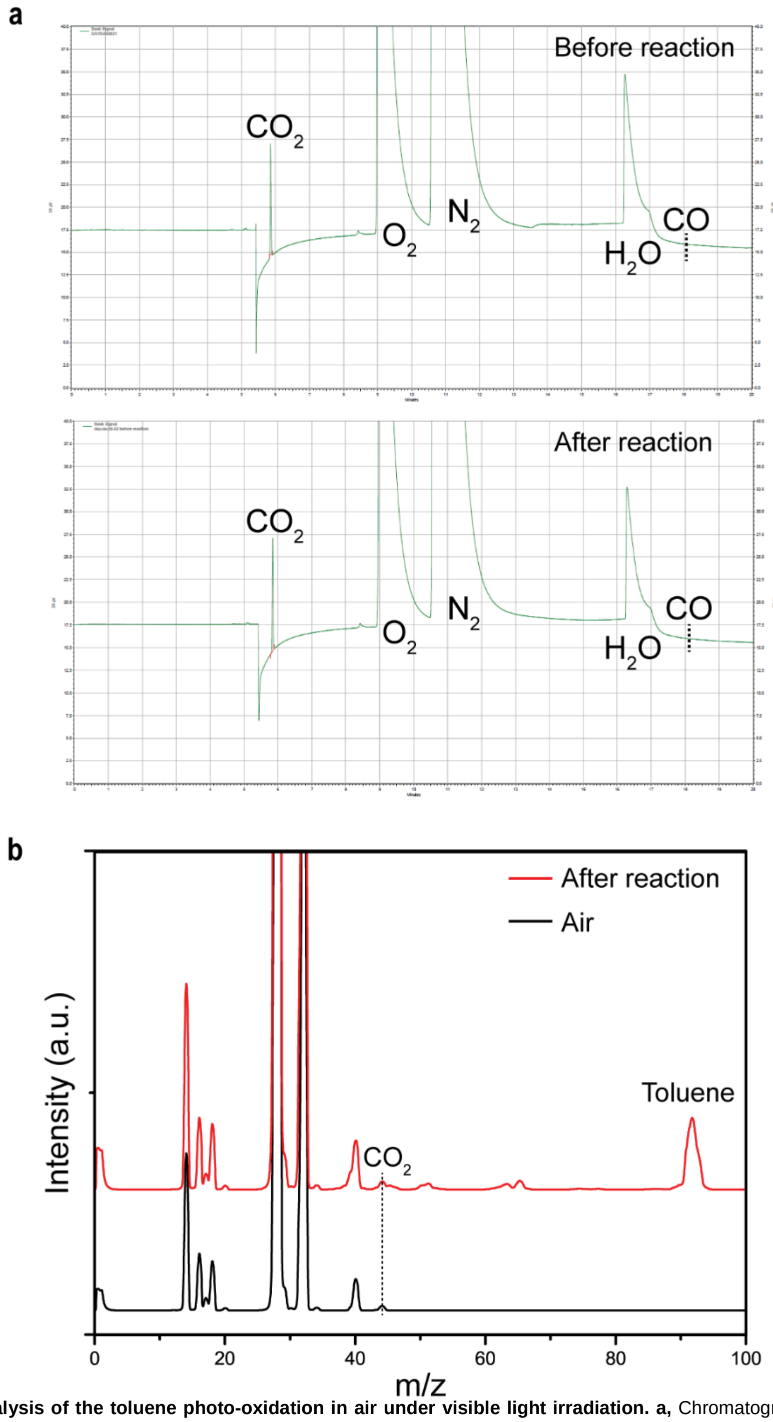

Figure S6. **Gas phase analysis of the toluene photo-oxidation in air under visible light irradiation. a,** Chromatograms of TCD signals from our online GC setup. **b,** MS signals from a mobile mass spectrometer connected to the reactor (red line indicates the measurement after reaction and black line means the clean air atmosphere used for reaction).

During the photo-oxidation of toluene in air under visible light irradiation, the gas phase was analysed by online GC setup and a mobile mass spectrometer. As shown in Figure S6a, the GC results show that before reaction a tiny amount of CO_2 was detected in the reaction atmosphere, which was due to the residual CO_2 in lab environment. After reaction, the CO_2 amount did not change and no CO was detected by a thermal conductivity detector (TCD) in GC setup. Due to the relatively lower sensitivity of TCD, the gas sample analysis was also performed on a mass spectrometer with a continuous mass-to-charge ratio (m/z) scan from 1 to 200, which did not detect the production of CO_2 after reaction as well (Figure S6b).

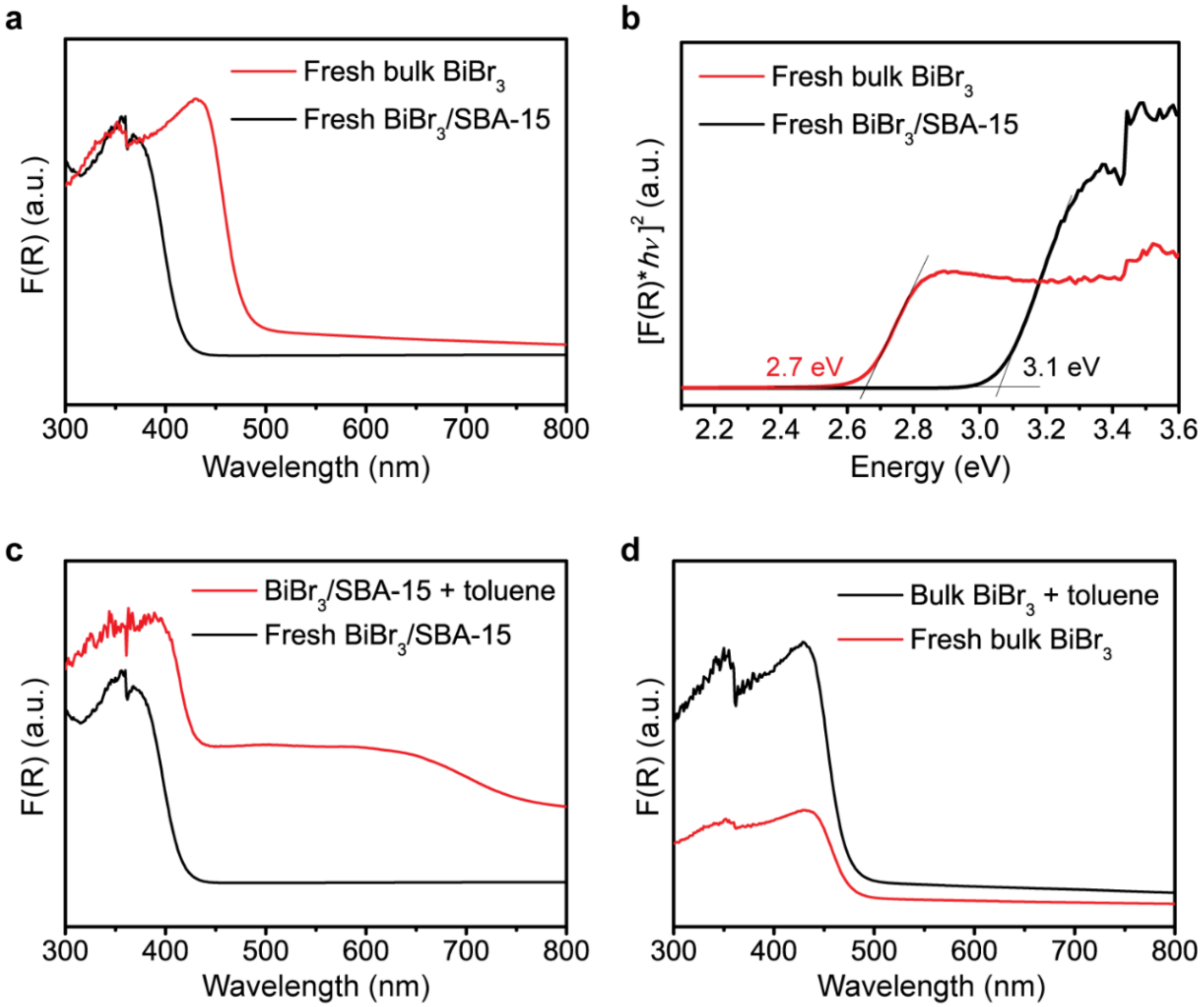

Figure S7. **UV-Vis DRS spectra of $BiBr_3$ based samples. a,** Light absorption and **b,** Tauc plots of 10 wt.% $BiBr_3$/SBA-15 and bulk $BiBr_3$. **c,** UV-Vis DRS spectra of $BiBr_3$/SBA-15 supported sample after toluene adsorption. **d,** UV-Vis DRS spectra of bulk $BiBr_3$ sample after toluene adsorption.

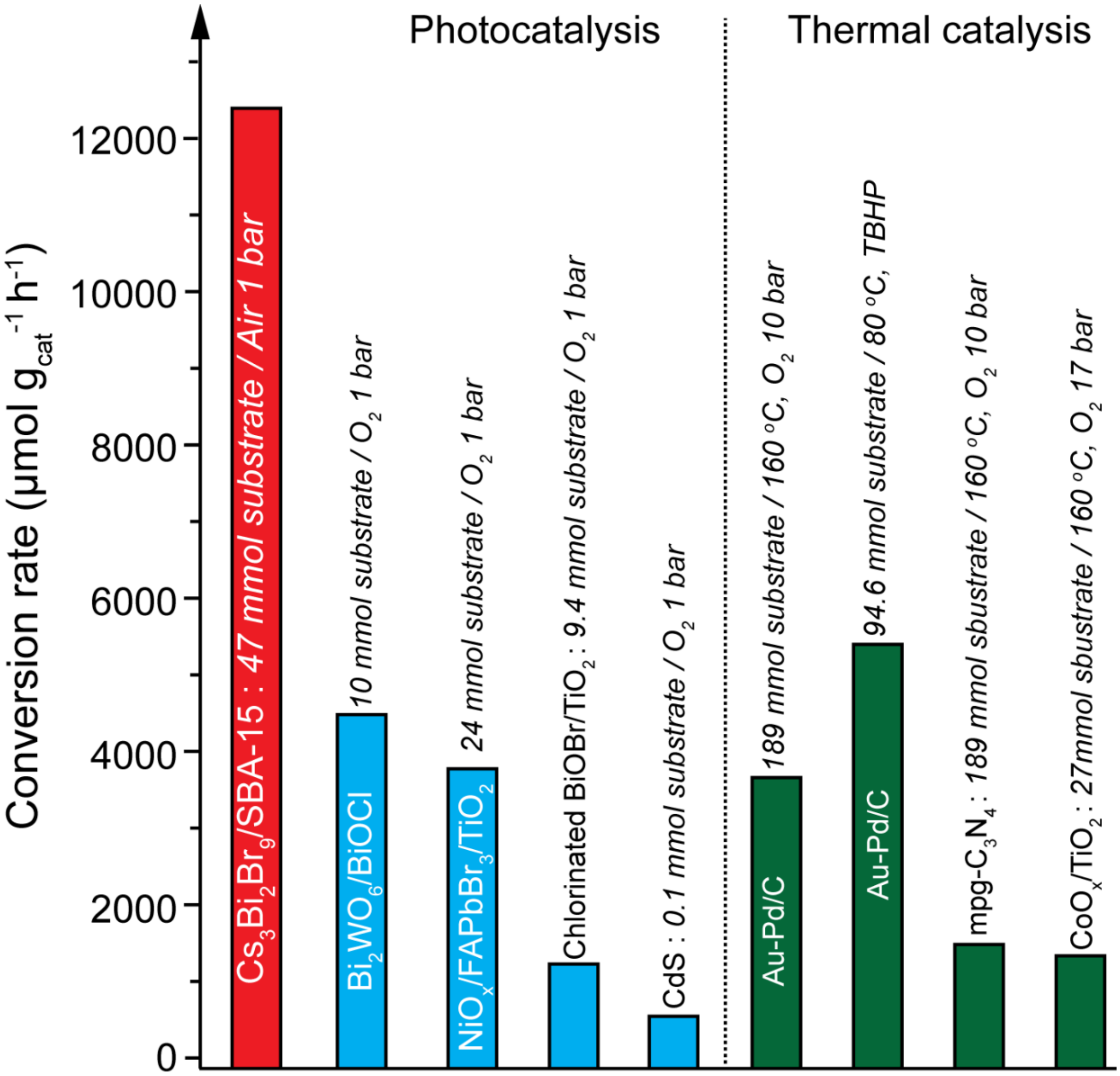

Figure S8. **Performance comparison with reported heterogenous catalysts for toluene oxidation including photocatalytic and thermal catalytic systems[17].**

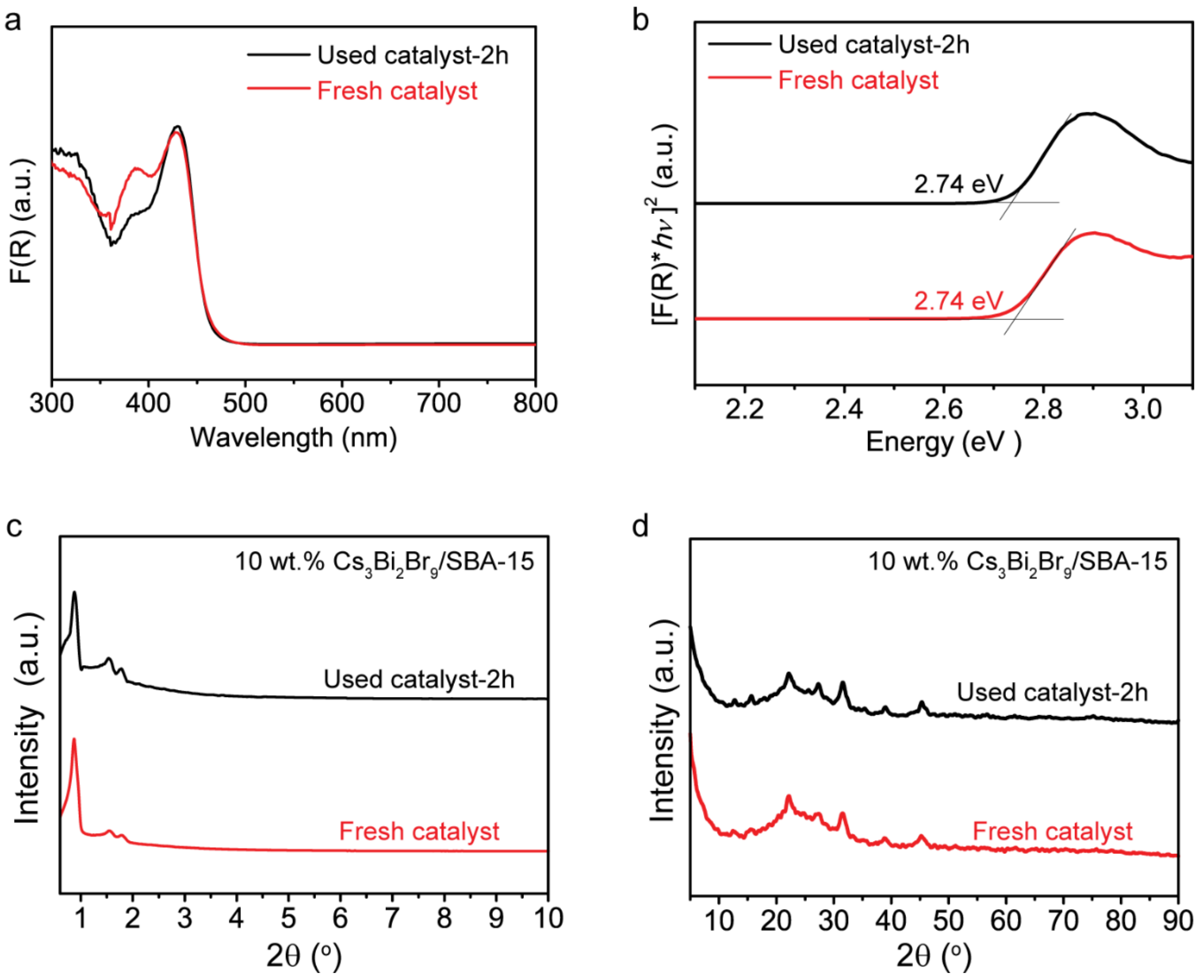

Figure S9. UV-Vis DRS (a, b) and XRD (c, d) results of the used and fresh 10 wt.% $Cs_3Bi_2Br_9$/SBA-15 sample.

Table S2. Content of the leached species in the liquid phase after reaction based on element analysis.

Liquid	Bi (ppm)	Cs (ppm)	Br (ppm)
Toluene	2	1	4

Table S3. The reaction results of photo-oxidation of different hydrocarbons over 10 wt.% $Cs_3Bi_2Br_9$/SBA-15 under visible light irradiation (≥ 420 nm)[a].

Entry	Hydrocarbon	Reaction time (h)	Conversion (%)[b]	Selectivity (%)[c]		Yield(%)[d]
				Aldehyde /ketone	Alcohol	
1	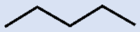	6	0.02	67	33	0.013
2	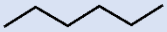	4	0.04	74	26	0.03
3	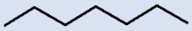	4	0.03	74	26	0.022
4	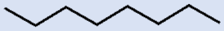	4	0.03	76	24	0.023
5[e]	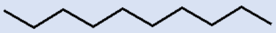	5	0.02	>99	-	0.02
6[e]	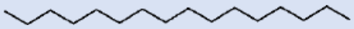	10	0.10	>99	-	0.10
7	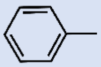	12	0.26	90	10	0.23
8	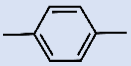	2	0.11	85	15	0.09
9	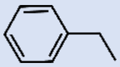	2	0.16	>99	-	0.16
10	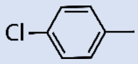	2	0.06	80	20	0.048
11[e]	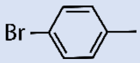	2	0.21	85	15	0.18
12[e]	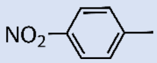	4	0.13	89	11	0.12

[a] Reaction conditions: 5 mL liquid hydrocarbon, 10 mg 10 wt.% $Cs_3Bi_2Br_9$/SBA-15 as photocatalyst, 1 bar air at 293 K under visible light (≥ 420 nm) irradiation. [b] Based on the total amount of produced ketones/aldehydes/alcohols determined by GC/MS. [c] Selectivity for aldehydes/ketones and alcohols, respectively. [d] Yield was calculated based on conversion and selectivity for carbonyl products. [e] Reaction temperature of 373 K due to higher melting points of substrates.

Table S4. Detailed oxygenated product distribution for the photo-oxidation of different aliphatic alkanes.

Hydrocarbon	Oxygenated product distribution (%)	
	Aldehyde/ketone	Alcohol
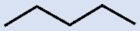	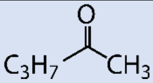 67	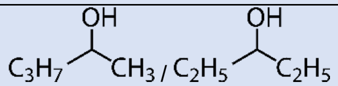 23 : 11
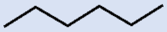	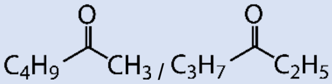 40 : 34	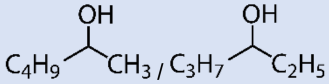 14 : 12
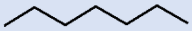	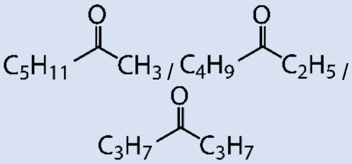 33 : 25 : 16	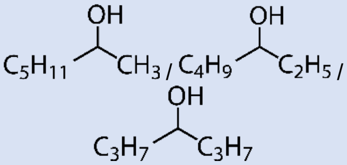 11 : 8 : 7
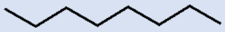	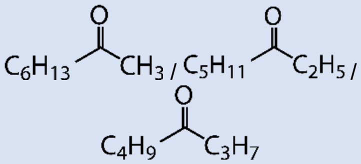 28 : 22 : 26	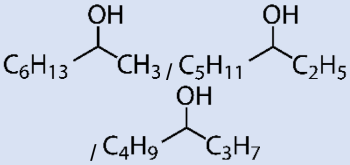 9 : 6 : 9
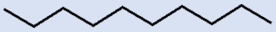	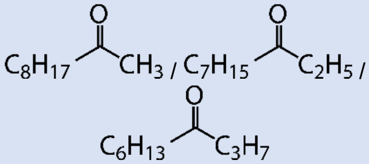 37 : 36 : 27	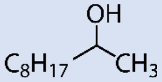 -
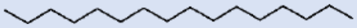	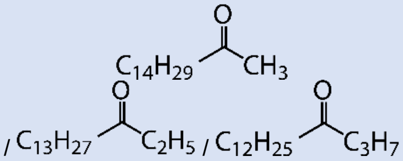 38 : 32 : 30	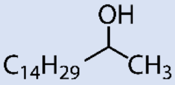 -

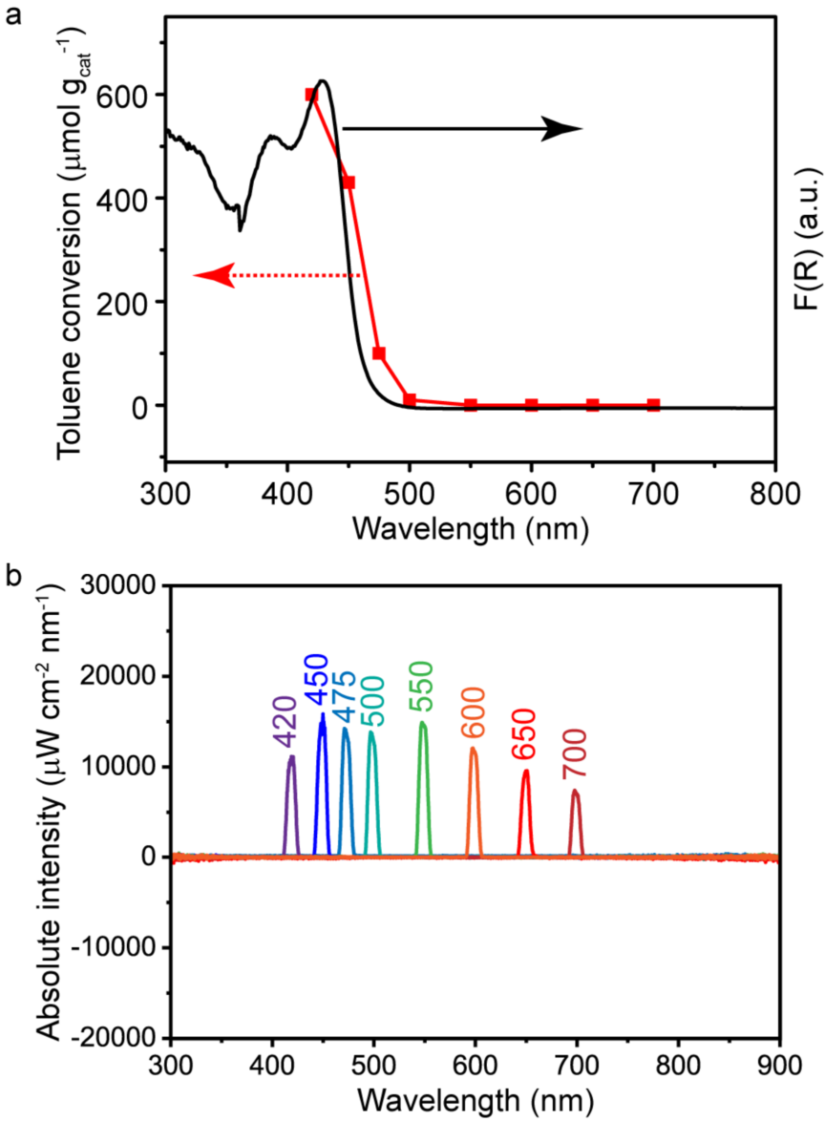

Figure S10. **Photocatalytic performance under monochromatic light irradiation. a,** Action spectrum. **b,** Light intensity of monochromatic light with different wavelengths at 420 nm, 475 nm, 450 nm, 500 nm, 550 nm, 600 nm, 650 nm and 700 nm, respectively.

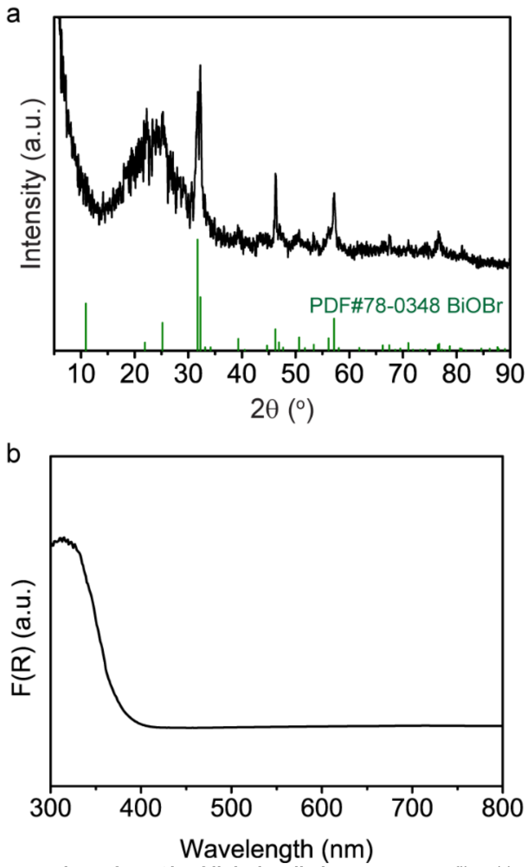

Figure S11. **Characterizations of the spent catalyst after 12h of light irradiation. a,** XRD profile with a reference (PDF#78-0348). **b,** UV-Vis DRS spectrum.

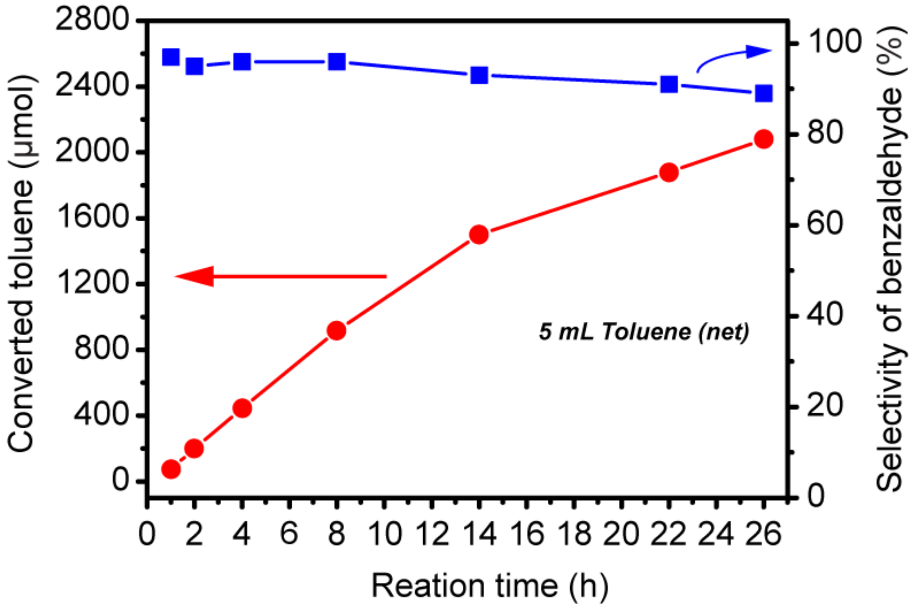

Figure S12. Time-dependent reaction performance for toluene photo-oxidation. Reaction conditions: 200 mg 10 wt.% $Cs_3Bi_2Br_9$/SBA-15 as photocatalyst, 200 mg Na_2SO_4 as desiccant, 5 mL toluene saturated with O_2, irradiated by UV-Vis light (200-1000 nm, 300 W Xe lamp)

Table S5. C(sp^3)-H bond dissociate energies (BDEs) of aromatic and aliphatic substrates.

Aromatics	C(sp^3)-H BDEs (kJ/mol)[a]
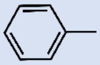	375.5 ± 5.0
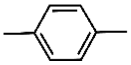	369.7
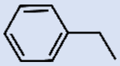	357.3± 6.3
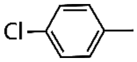	371.3
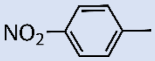	373.4
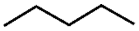	415.1[b]
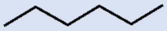	410.0[b]
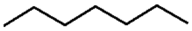	410.0[b]

[a] Most recommended experimental values cited from ref.[18] and [19]. [b] Secondary C-H bond

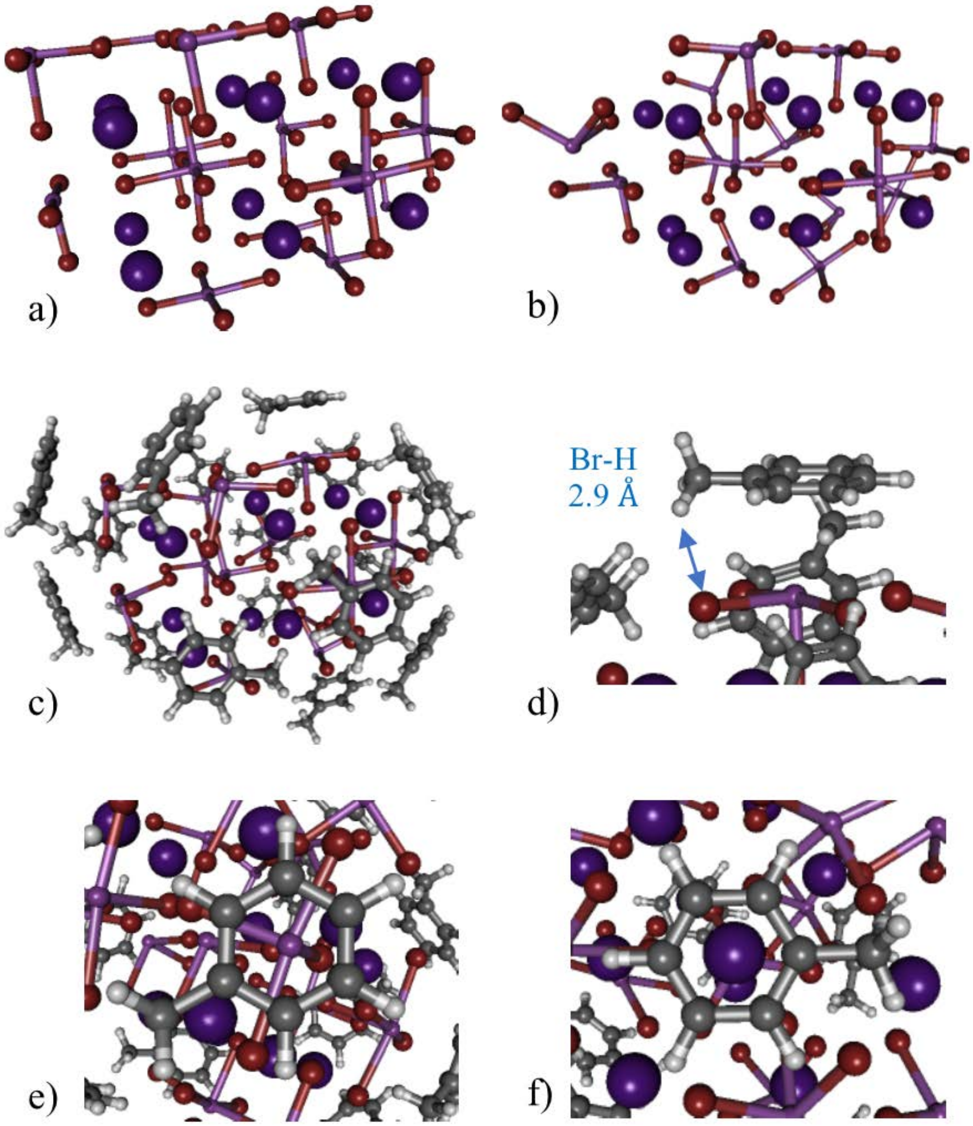

Figure S13. **a,** $Cs_{12}Bi_{14}Br_{54}$ cluster from crystal structure of $Cs_3Bi_2Br_9$ and **b,** optimized at the PBE-D3/def2-svp level. **c,** Optimized geometry of the $Cs_{12}Bi_{14}Br_{54}$ cluster with 17 toluene molecules with focus on **d,** a typical Br-H geometry, **e,** a toluene Bi interaction and **f,** a toluene Cs interaction.

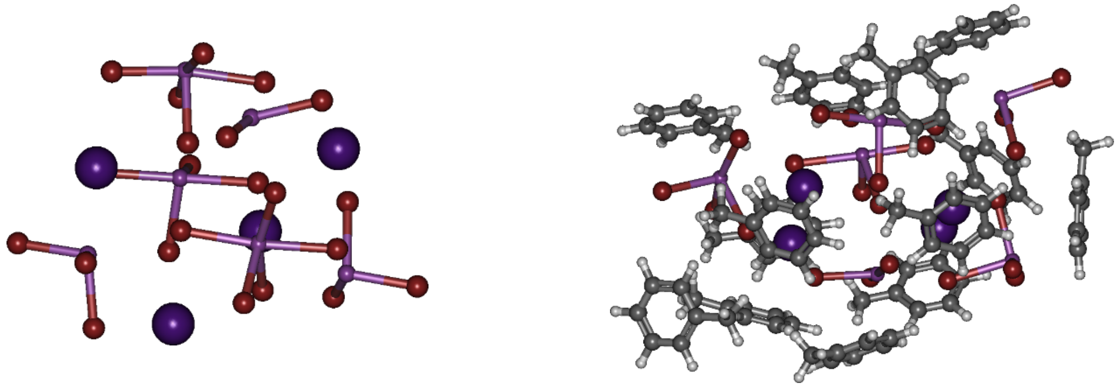

Figure S14. Optimized $Cs_4Bi_6Br_{22}$ and $Cs_4Bi_6Br_{22}$ + 17 toluene molecules structures.

In order to gain insight into the interaction and the reactivity between organic molecules and $Cs_3Bi_2Br_9$ perovskite nanoparticles, cluster models were designed and properties were calculated using density functional theory (DFT). For the perovskite cluster models, we imposed stoichiometries leading to a neutral system according to the formal charges the atoms (*i.e.*, Cs^{I}, Bi^{III}, Br^{-I}) and used the crystal structure as starting point for the geometry. Several models have been considered, changing the surface termination and the size of the cluster. We found that Bi-rich surfaces lead to structurally stable and neutral clusters upon geometry optimization. The model that have been chosen are small enough to allow for electronic structure calculations but also large enough to reach the size range of the experiment (~1.5 nm, see Figure 4b in main text and Figure S13b). Notably, the built model as a surface-dominant cluster is mainly designed to simulate the possible surface environment of $Cs_3Bi_2Br_9$ nanoparticles. Additionally, a smaller cluster has been studied (~1.0: 0.9: 0.7 nm for size in three dimensions, see Figure S14) to observe whether some of the size effects measured experimentally could be reproduced and rationalized. Meanwhile, in Table S1 the sample with a lower loading of halide perovskite has a lower composition ratio of Cs: Bi and Bi: Br (*e.g.*, Cs: Bi: Br = 3.0: 2.2: 7.6 for the 5 wt.% sample and 3.0: 1.8: 9.8 for the 40 wt.% sample). Clearly, the perovskite material in the sample with lower loading (*e.g.*, 5 wt.%) are dominantly small $Cs_3Bi_2Br_9$ nanoparticles (2-5 nm, Figure 1b in main text), while the sample with high-level loading (40 wt.%) consists of large particles of $Cs_3Bi_2Br_9$ (>100 nm, Figure 1k and 1l in main text). It is known that the composition of nanoparticles involves more surface portions when the size of nanoparticle gets smaller. Even though the trend variation based on experimental data in Table S1 appears slight, we may assume that one possible surface environment of small $Cs_3Bi_2Br_9$ nanoparticles could be bismuth-rich with less Cs and Br atoms. Moreover, the smaller cluster (Figure S14) with a higher surface-to-bulk ratio presents even less Cs content, which could also support our guess. Upon optimization, the core of the particle remains rather unchanged while the structure of the surface atoms, namely Bi and Br, relaxes leading to tetrahedral $BiBr_3$ and square planar $BiBr_5$ arrangements.

Subsequently, toluene molecules were placed at the surface of the cluster and the systems were then fully optimized (see Figure 4c-d in main text and Figure S13c-f), resulting in a coordination of the phenyl moiety on top of either a Bi or Cs atom. According to previous studies, this interaction with Bi atoms is dominated by dispersion but with a significant contribution of charge transfer from the π system to a σ* orbital of the Bi atom[20]. Here, a total charge transfer from the toluene molecules to the perovskite of about 1.5 electrons is found in the case of the large cluster (0.9 for the smaller cluster), leading to a partially reduced cluster. Additionally, this interaction also brings the reactant's CH_3 groups close to surface Br atoms (see Figure 4d in main text and Figure S13d) which hints at a potential reaction site.

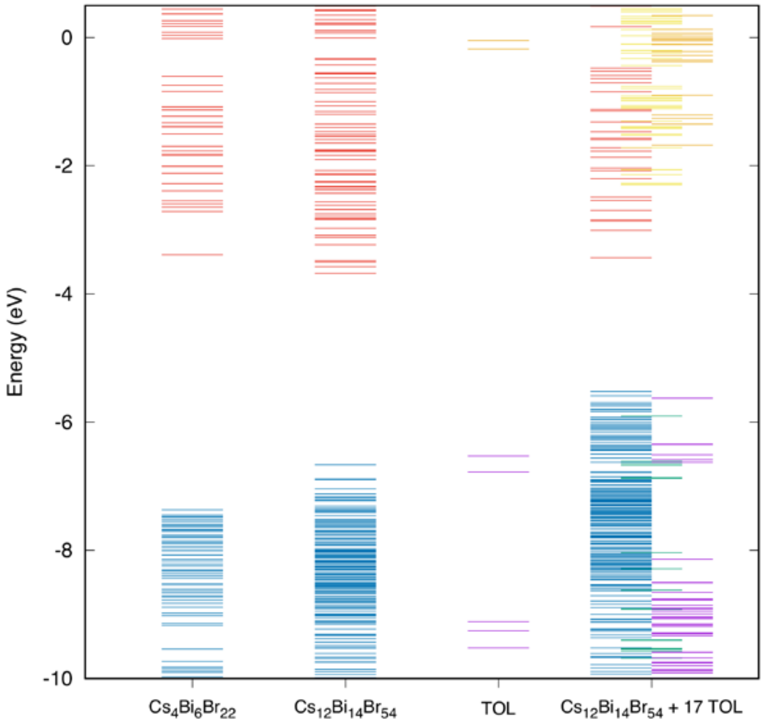

Figure S15. Molecular orbital energies of the $Cs_4Bi_6Br_{22}$ and $Cs_{12}Bi_{14}Br_{54}$ clusters, toluene (TOL) and the $Cs_{12}Bi_{14}Br_{54}$ cluster with 17 toluene molecules calculated at the B3LYP-D3/def2-svp level. Blue (red) lines: occupied (unoccupied) MOs of $Cs_{12}Bi_{14}Br_{54}$; purple (orange) lines: occupied (unoccupied) MOs of toluene; green (yellow) lines: occupied (unoccupied) mixed MOs of the perovskite of the toluene molecules

Figure S15 shows the calculated molecular orbital energies of $Cs_4Bi_6Br_{22}$ and $Cs_{12}Bi_{14}Br_{54}$ clusters, C_7H_8 and the $Cs_{12}Bi_{14}Br_{54}$ + $(C_7H_8)_{17}$. Increasing the size of the cluster model decreases the HOMO-LUMO gap, which correlates with experiments. Upon interaction with toluene, an increase of the energy of the occupied orbitals of the perovskite cluster is predicted. This can be explained by the charge transfer from the toluene molecules to the cluster.

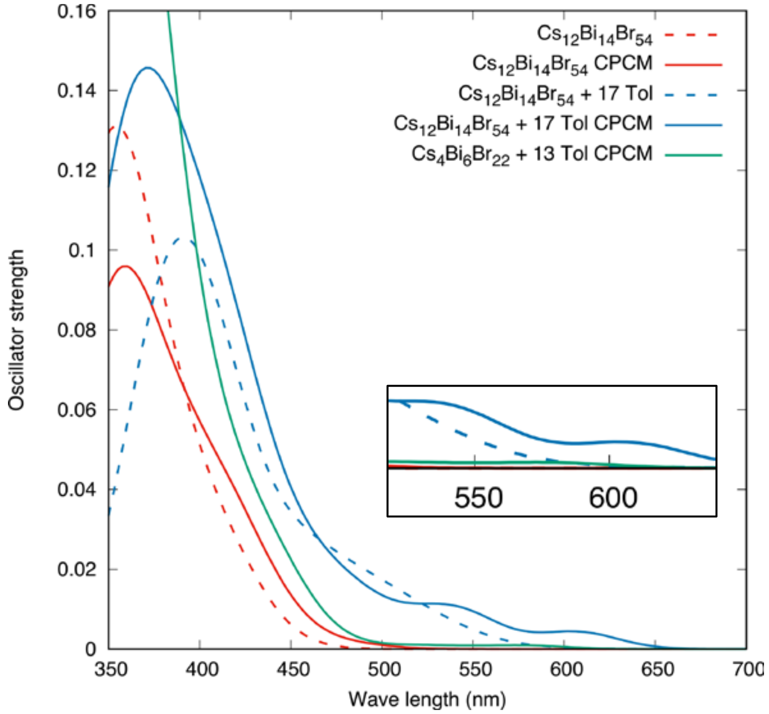

Figure S16. Calculated UV-Vis absorption spectra of the $Cs_{12}Bi_{14}Br_{54}$ cluster alone and with 17 toluene molecules (red and blue lines, respectively) without and with a CPCM model of toluene (dashed and plain lines, respectively), as well as the $Cs_4Bi_6Br_{22}$ cluster with 13 toluene molecules with CPCM model of toluene (green line) calculated at the B3LYP-D3/def2-svp level.

Our calculations of UV-Vis absorption spectra reproduce the main features of the experimental UV-Vis spectra as shown in Figure S16. The presence of toluene molecules at the surface of the perovskite cluster yields absorption peaks from about 500 nm to 620 nm, which is not observed when only including an implicit model of toluene (CPCM). In the case of the smaller cluster ($Cs_4Bi_6Br_{22}$) with 13 toluene molecules, only very few and weak absorption peaks can be found in this region. One can anticipate this low energy band to increase in intensity and decrease in energy as the size of the system increases.

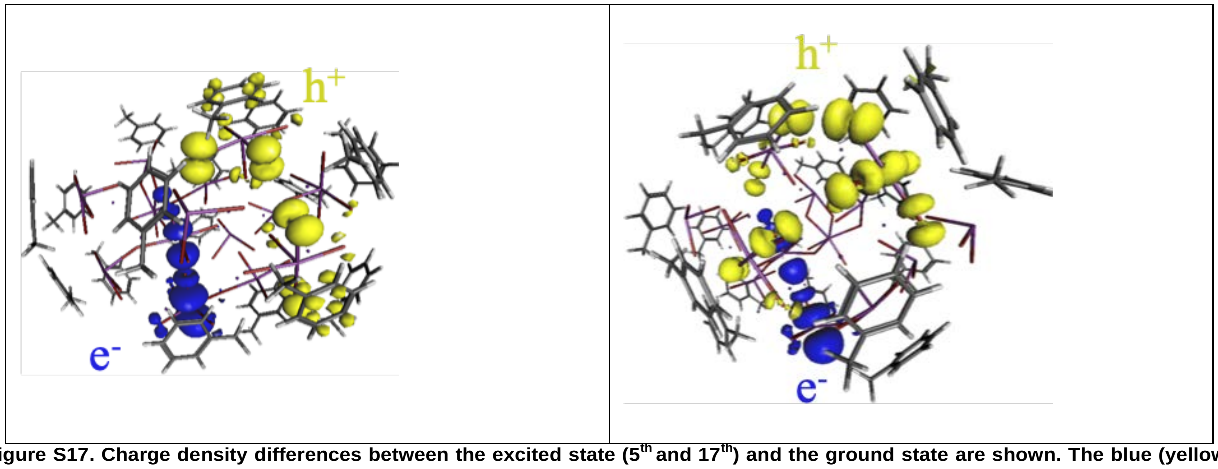

Figure S17. Charge density differences between the excited state (5th and 17th) and the ground state are shown. The blue (yellow) surfaces correspond to the increase of electron (or decrease of hole) density upon excitation.

The states in this region are characterized by weak absorption intensities and excitations corresponding to an electron transfer from mostly the lone pairs of Br atoms with a small contribution of π system of toluene molecules to an empty orbital of a Bi atom, as shown in the Figure S17.

Table S6. Frontier molecular orbitales of $Cs_{12}Bi_{14}Br_{54}$ + 17 toluene molecules calculated at the B3LYP-D3/def2-SVP CPCM level.

HOMO-8 (1450)	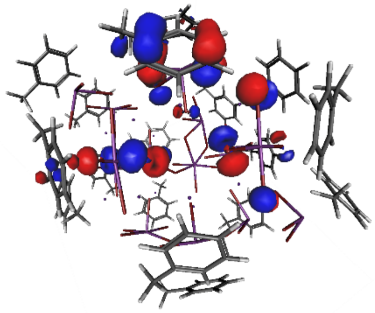
HOMO-7 (1451)	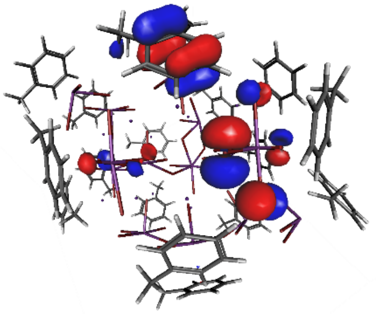
HOMO-6 (1452)	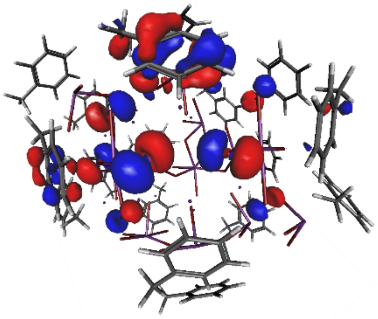
HOMO-5 (1453)	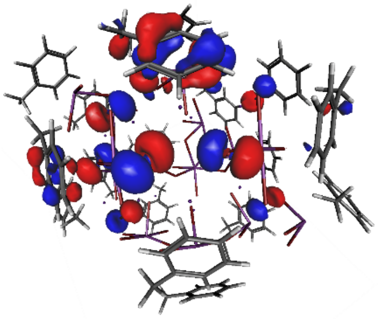
HOMO-4 (1454)	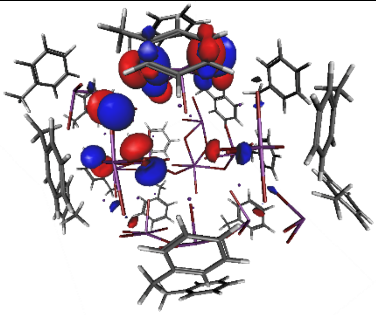

HOMO-3 (1455)	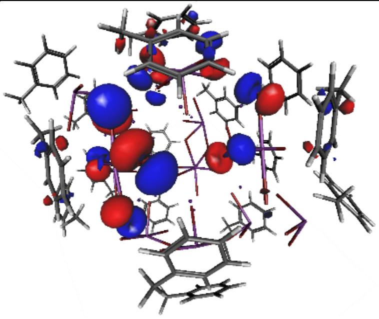
HOMO-2 (1456)	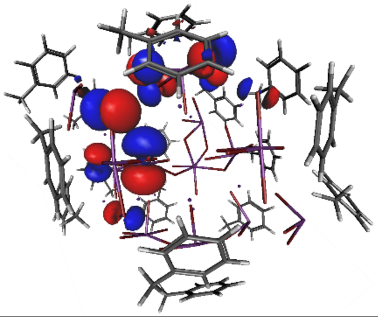
HOMO-1 (1457)	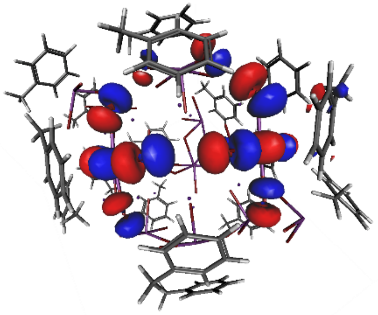
HOMO (1458)	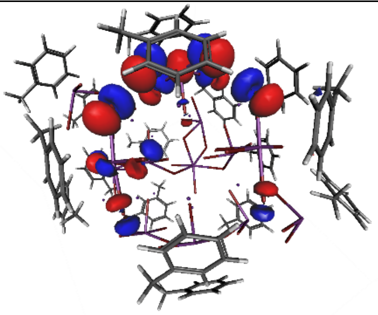
LUMO (1459)	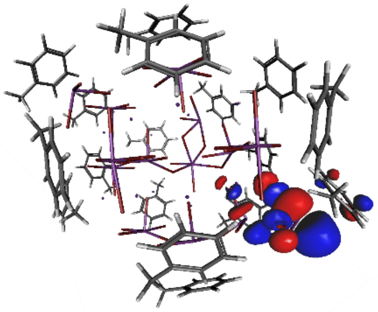

LUMO+1 (1460)	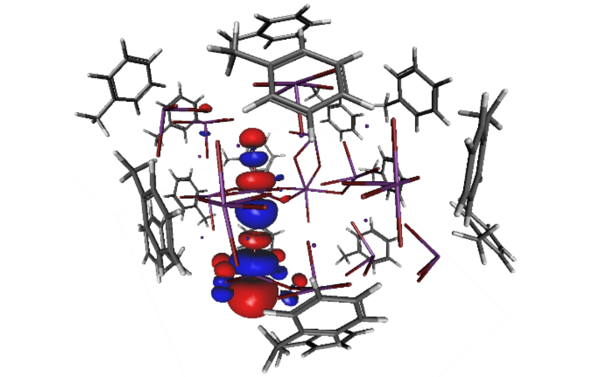

Excited state 5 (2.044 eV)	
Mono-excitations	Weight (coefficient)
1455 -> 1460	0.016058 (c= 0.12672079)
1456 -> 1460	0.010067 (c= 0.10033527)
1457 -> 1460	0.705509 (c= 0.83994564)
1458 -> 1460	0.253990 (c= -0.50397415)

Excited state 17 (2.284 eV)	
Mono-excitations	Weight (coefficient)
1450 -> 1460	0.016472 (c= 0.12834383)
1452 -> 1460	0.651773 (c= 0.80732432)
1453 -> 1460	0.244161 (c= 0.49412654)
1454 -> 1460	0.058035 (c= -0.24090399)

Table S7. Vertical excitations of $Cs_{12}Bi_{14}Br_{54}$ + 17 toluene molecules calculated at the B3LYP-D3/def2-SVP CPCM level.

State	Energy (cm^{-1})	Wavelength (nm)	Oscillator strength
1	14461.4	691.5	4.883E-06
2	13917.8	718.5	4.6362E-05
3	16295.8	613.7	0.00088029
4	16684.8	599.3	0.00023201
5	16485.6	606.6	0.00293149
6	15744.6	635.1	4.3101E-05
7	15258.2	655.4	1.5532E-05
8	16277.9	614.3	7.475E-06
9	15957.1	626.7	2.934E-06
10	17746.2	563.5	0.00028535
11	16355.8	611.4	6.5861E-05
12	17919.4	558.1	0.00068757
13	17334.9	576.9	2.8363E-05
14	18325.6	545.7	0.00019955
15	15980.2	625.8	4.2532E-05
16	18444.6	542.2	0.00139145

17	18422.8	542.8	0.00424381
18	17583	568.7	4.2969E-05
19	18696.9	534.8	5.7313E-05
20	18075.4	553.2	0.00022846
21	19204.6	520.7	0.00090555
22	17282.3	578.6	0.00033022
23	18565.9	538.6	0.00017339
24	18088.4	552.8	2.1913E-05
25	18975.7	527	0.00017127
26	20158.9	496.1	0.00183641
27	19627.1	509.5	0.00043198
28	18407	543.3	0.00018303
29	18829.8	531.1	0.00035272
30	19799.9	505.1	3.1231E-05
31	18822.2	531.3	1.3132E-05
32	18956.4	527.5	0.0008378
33	18293.3	546.6	8.6766E-05
34	18477.7	541.2	3.5095E-05
35	20020.8	499.5	0.00010118
36	19514.1	512.4	0.00100871
37	19984.6	500.4	7.8576E-05
38	21086.5	474.2	0.00031981
39	20468.3	488.6	7.3235E-05
40	18805.6	531.8	2.7683E-05
41	20042.8	498.9	5.7977E-05
42	21208.2	471.5	0.00117291
43	21150.7	472.8	0.00045744
44	20304.6	492.5	0.00049985
45	20024.5	499.4	0.00004963
46	19930	501.8	0.00028051
47	20663.9	483.9	0.00158642
48	19694.7	507.8	0.00010611
49	21030.6	475.5	0.00110845
50	19758.9	506.1	8.728E-06
51	20344.7	491.5	0.00019322
52	20301.4	492.6	0.00016818
53	21279.6	469.9	0.00039375
54	21172.4	472.3	0.00037628
55	21772.9	459.3	0.00320794

56	21442.2	466.4	0.00098407
57	21060.4	474.8	0.00078874
58	19326	517.4	0.00008974
59	21039.1	475.3	0.00084322
60	20938.7	477.6	0.00105252
61	21031.7	475.5	6.2143E-05
62	20997	476.3	7.6111E-05
63	21031.6	475.5	4.1968E-05
64	21387.3	467.6	0.00037763
65	20457.3	488.8	2.0886E-05
66	21894.9	456.7	0.00019241
67	21029.2	475.5	0.00030311
68	14942.4	669.2	9.192E-06
69	21417.4	466.9	0.00016896
70	19958.5	501	4.9787E-05
71	20981.7	476.6	6.1417E-05
72	21670.2	461.5	0.00050777
73	20897.6	478.5	0.00014122
74	19030.9	525.5	0.00054536
75	21810.1	458.5	7.4437E-05
76	23854.1	419.2	0.00241063
77	21127.8	473.3	0.00020039
78	24035.5	416.1	0.0019131
79	22749	439.6	0.0007951
80	22930.9	436.1	0.00068909
81	20373	490.8	4.7912E-05
82	22560.2	443.3	0.00281005
83	22328.8	447.9	0.0009681
84	21817.1	458.4	0.00011212
85	22582.5	442.8	0.00277139
86	24876.6	402	0.00220116
87	23022.5	434.4	0.00088872
88	22396.5	446.5	4.4738E-05
89	23399.5	427.4	0.00133235
90	23140.9	432.1	0.00086028
91	22557.6	443.3	0.00070091
92	21274.7	470	8.5503E-05
93	22998.4	434.8	0.00039497
94	21408.9	467.1	1.3544E-05

95	21319.3	469.1	1.8262E-05
96	22607	442.3	0.0006605
97	22576	442.9	0.00012062
98	22761.6	439.3	0.00064954
99	21864.7	457.4	8.9152E-05
100	22742	439.7	0.00029699
101	21869.5	457.3	2.2699E-05
102	23367.5	427.9	0.00039054
103	23024.4	434.3	4.5854E-05
104	23251.1	430.1	0.00255922
105	23104.7	432.8	0.00020933
106	18795.3	532	0.00019745
107	22266.8	449.1	0.0000633
108	23368.9	427.9	0.00049206
109	24540.6	407.5	0.00316831
110	23568.8	424.3	0.00011233
111	22357	447.3	0.00013958
112	23552	424.6	0.00190258
113	23317.3	428.9	0.00031915
114	23752.9	421	0.00029655
115	23906.3	418.3	0.00073354
116	23936.6	417.8	0.00018584
117	23670.3	422.5	0.00026574
118	22287.9	448.7	4.2006E-05
119	23461.7	426.2	0.00170545
120	22079.9	452.9	4.0297E-05
121	22993.2	434.9	0.00063285
122	22886.2	436.9	0.00075034
123	23924.4	418	0.00016076
124	24229.3	412.7	0.00165909
125	22674	441	1.4442E-05
126	23956.5	417.4	0.00061193
127	22900	436.7	0.00055977
128	22782	438.9	3.8622E-05
129	23726.7	421.5	7.8794E-05
130	23383	427.7	0.00049105
131	22597.8	442.5	4.893E-06
132	23633.9	423.1	0.00020323
133	26057.5	383.8	0.00234927

134	25158.8	397.5	0.00255746
135	26199.7	381.7	0.00135584
136	24449	409	0.00306365
137	23481	425.9	0.00056228
138	24348	410.7	0.00042798
139	24315.9	411.3	0.00108343
140	25086.2	398.6	0.00033181
141	25140.5	397.8	0.00172402
142	24291.7	411.7	0.00031078
143	23803.9	420.1	0.00303029
144	24011.2	416.5	0.00033377
145	24223	412.8	0.00322218
146	22077.6	452.9	0.00015764
147	24431.9	409.3	0.00906173
148	24254.8	412.3	0.00047093
149	24820.4	402.9	0.00029557
150	24769.1	403.7	0.00088864
151	24261.4	412.2	0.00120527
152	24399.2	409.8	0.00034823
153	24203.2	413.2	0.00086358
154	25190.4	397	0.00120731
155	23251.1	430.1	0.00010246
156	24405.5	409.7	5.2961E-05
157	23996	416.7	0.00012305
158	24491.8	408.3	0.00685453
159	24527.3	407.7	8.5141E-05
160	24932.9	401.1	0.00019032
161	26535.3	376.9	7.1148E-05
162	25416.2	393.5	3.2776E-05
163	25259.1	395.9	0.00021681
164	24921.1	401.3	0.00049995
165	24516	407.9	8.252E-06
166	24951	400.8	0.00067145
167	23759.7	420.9	0.00055525
168	23600.6	423.7	0.00015198
169	23392.3	427.5	8.0483E-05
170	25161.9	397.4	0.00090539
171	25455.3	392.8	0.00020307
172	24098.9	415	0.00018163

173	23563.1	424.4	5.7612E-05
174	25039	399.4	0.000334
175	25416.3	393.4	0.00511483
176	25325	394.9	0.00229107
177	24426.7	409.4	0.00020152
178	24738.9	404.2	0.00039064
179	24346.6	410.7	0.00062966
180	24618.2	406.2	0.0001314
181	25506.3	392.1	0.00042285
182	24728.1	404.4	0.00040646
183	25745.3	388.4	0.00090837
184	25780.7	387.9	0.00160158
185	25166.6	397.4	0.00010535
186	25368.6	394.2	0.00015566
187	25804.1	387.5	0.00020061
188	26252.3	380.9	0.00192229
189	25552.3	391.4	0.00043507
190	25340.5	394.6	0.00019597
191	26726.8	374.2	0.00728891
192	24886.2	401.8	6.714E-06
193	23762.6	420.8	3.7176E-05
194	26042.1	384	8.8912E-05
195	26317.4	380	0.00019222
196	26601	375.9	0.00150335
197	26494.7	377.4	0.00039183
198	25927.2	385.7	0.00049614
199	26278.5	380.5	0.00029287
200	26018.7	384.3	0.00010217
201	25271.4	395.7	0.00060136
202	25078.2	398.8	3.8107E-05
203	26250.6	380.9	0.00018329
204	26882	372	0.00516255
205	26155.8	382.3	0.00100778
206	26392.6	378.9	0.00239775
207	25301.3	395.2	0.00012381
208	25156.5	397.5	0.00042589
209	21626.1	462.4	4.2742E-05
210	27627.6	362	0.0026084
211	26144.6	382.5	0.00034377

212	25882.6	386.4	0.00069191
213	25815.6	387.4	0.00033319
214	25236.1	396.3	0.00088939
215	26308.7	380.1	0.00112153
216	23671.6	422.4	8.7058E-05
217	25570.6	391.1	0.00099537
218	25553.6	391.3	0.00031948
219	26787	373.3	0.00200664
220	25891.9	386.2	0.00081069
221	26797.6	373.2	0.00071243
222	27077.9	369.3	0.0018955
223	26112.1	383	0.00145681
224	26222.6	381.4	0.00163514
225	27951.1	357.8	0.00771646
226	25853.3	386.8	0.00042207
227	27818.2	359.5	0.00301369
228	25862.8	386.7	0.00024979
229	23094.4	433	0.00030544
230	24149	414.1	0.00023739
231	24078.2	415.3	0.00043206
232	26730	374.1	0.00055367
233	27529.4	363.2	0.00015634
234	26993.5	370.5	0.00029028
235	26158.7	382.3	0.00312693
236	27872.2	358.8	0.00143078
237	26865.9	372.2	0.00183729
238	27253.7	366.9	0.00202578
239	25694.7	389.2	2.8003E-05
240	26657	375.1	0.00012444
241	27663.4	361.5	0.00418595
242	26554.1	376.6	0.00057178
243	26333	379.8	0.00141537
244	27126.1	368.6	0.0021828
245	26054.9	383.8	0.00068775
246	26302.3	380.2	0.00091175
247	26655.6	375.2	0.00055027
248	26917.1	371.5	0.0004427
249	26483	377.6	0.00022468
250	27357.1	365.5	0.00180151

251	25709.3	389	0.0006848
252	27088.1	369.2	0.00056107
253	27196.3	367.7	0.00103114
254	26865.5	372.2	0.00075805
255	27908	358.3	0.00313072
256	28398.1	352.1	0.00219303
257	26832.3	372.7	0.00061677
258	26379	379.1	0.00087714
259	25007.6	399.9	0.00010184
260	26332.3	379.8	0.00029343
261	26780.9	373.4	0.00035289
262	26494.3	377.4	0.00027045
263	26934.3	371.3	0.00226927
264	26512.9	377.2	0.00090923
265	26325	379.9	0.00077422
266	26530.5	376.9	0.00013509
267	26719.3	374.3	0.00064025
268	25726.3	388.7	0.00057374
269	26372.4	379.2	7.4195E-05
270	27947.9	357.8	0.0008598
271	27346.1	365.7	0.00129081
272	25666.8	389.6	0.00001797
273	27400.3	365	0.00053283
274	27636.4	361.8	0.00010888
275	27857.2	359	0.00223249
276	26365.6	379.3	0.00195676
277	27314.9	366.1	0.0004869
278	26309.6	380.1	0.00032089
279	28835.7	346.8	0.00940176
280	25815.6	387.4	0.00025258
281	26739.6	374	0.00157588
282	26972.9	370.7	0.00033757
283	28799.2	347.2	0.00131923
284	27188	367.8	0.00107836
285	26760.4	373.7	0.00040079
286	28565.7	350.1	0.00067012
287	26811	373	0.00013875
288	27205.8	367.6	0.00054829
289	27918.6	358.2	0.00248938

290	29120	343.4	0.00340305
291	26822.9	372.8	0.00055855
292	28557.5	350.2	0.02029886
293	28737.3	348	0.00582022
294	27348.6	365.6	0.00017855
295	27637.3	361.8	0.00027593
296	27658.7	361.6	0.00128591
297	28566.7	350.1	0.00603362
298	27735.7	360.5	0.00062348
299	27379	365.2	0.00034615
300	21715	460.5	0.00032976

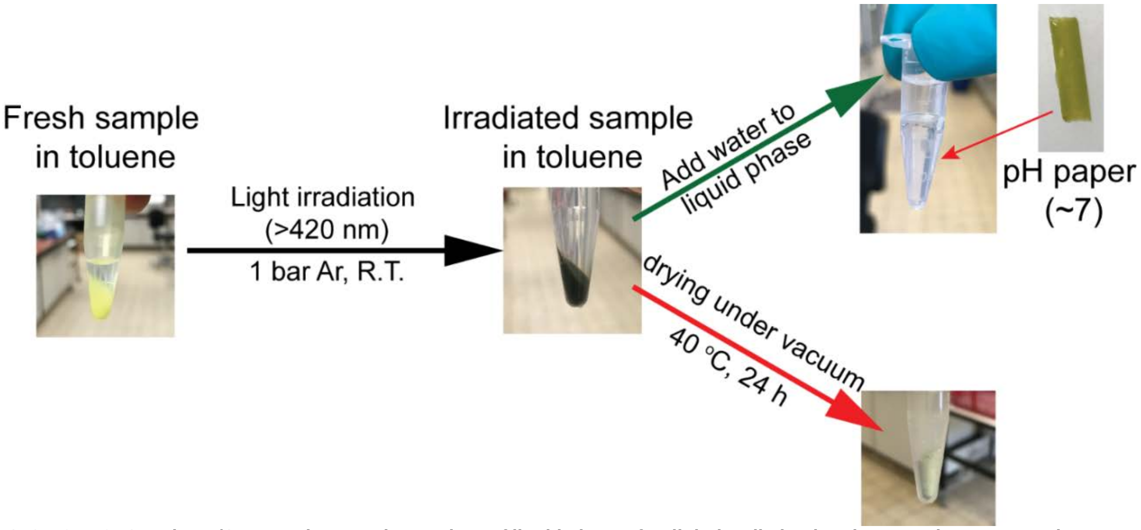

Figure S18. **10 wt.% $Cs_3Bi_2Br_9$/SBA-15 photocatalyst and pH of liquid phase after light irradiation in toluene under Ar atmosphere.**

a

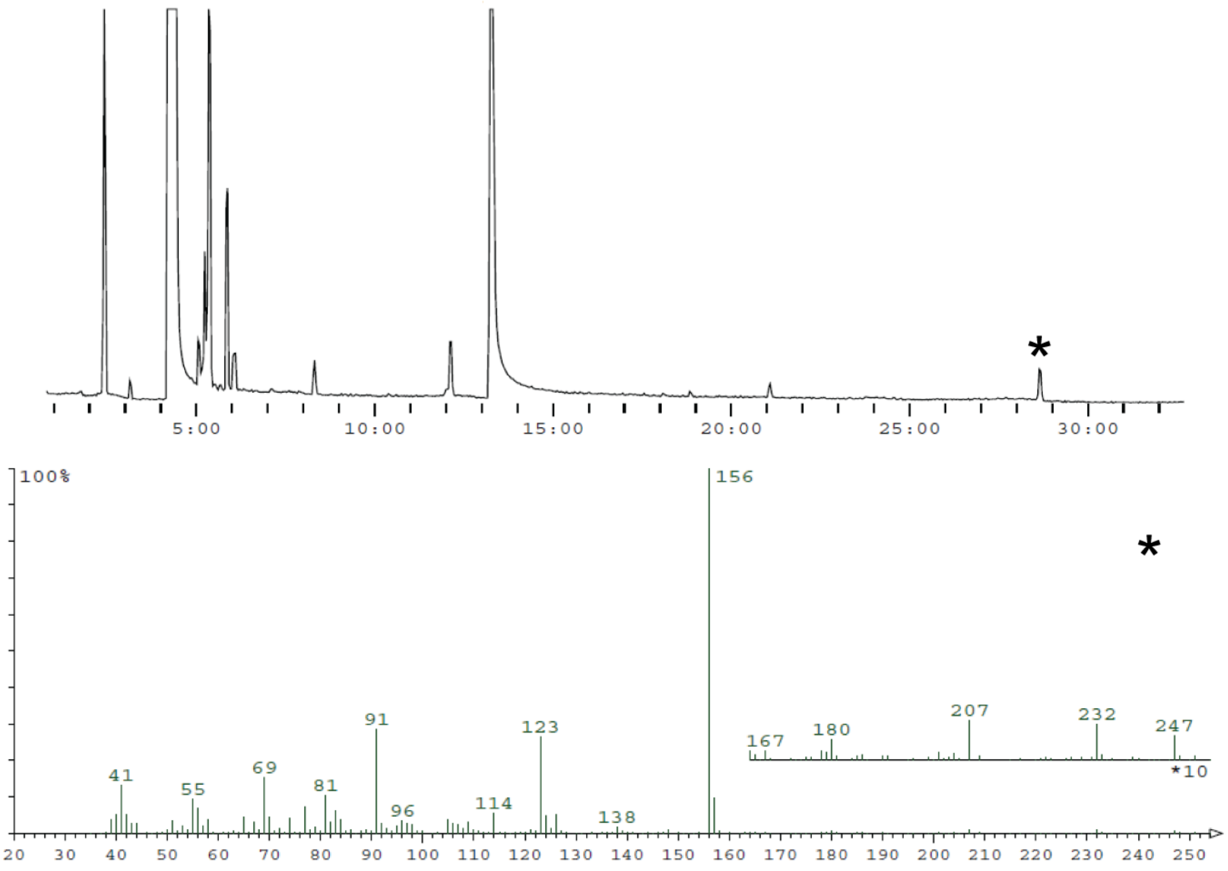

b

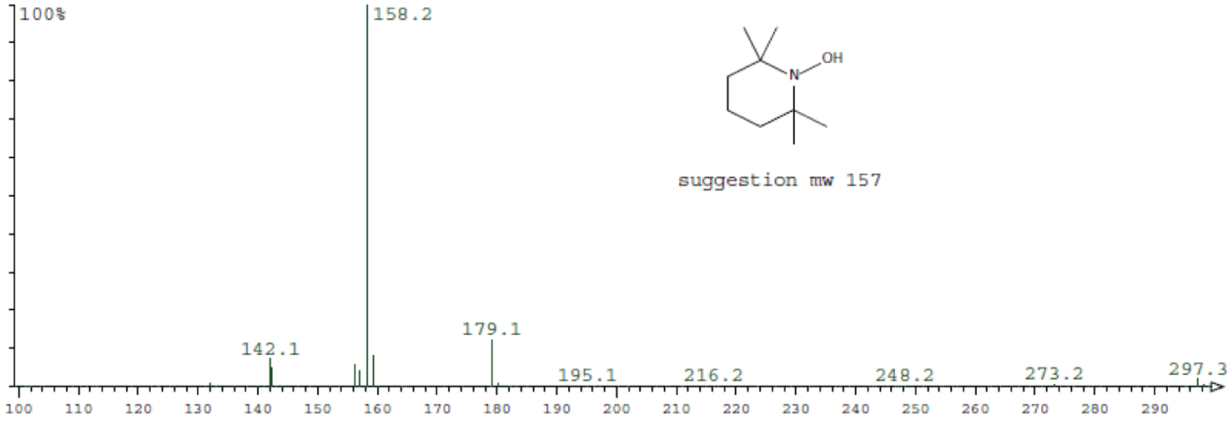

Figure S19. **a,** TEMPO-benzyl radical adduct compound (indicated by *) detected by GC/MS. **b,** TEMPO-H identified by electrospray ionization-MS.

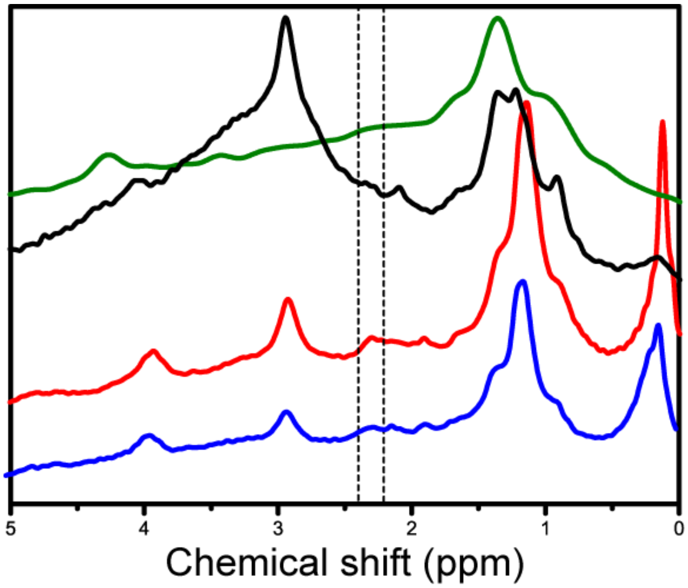

Figure S20. Solid-state ^{1}H nuclear magnetic resonance spectroscopy (NMR) analysis of the clean (black line), irradiated (red line) and air-heated (blue line) bulk $Cs_3Bi_2Br_9$ samples with $NaNO_3$ as reference (green line).

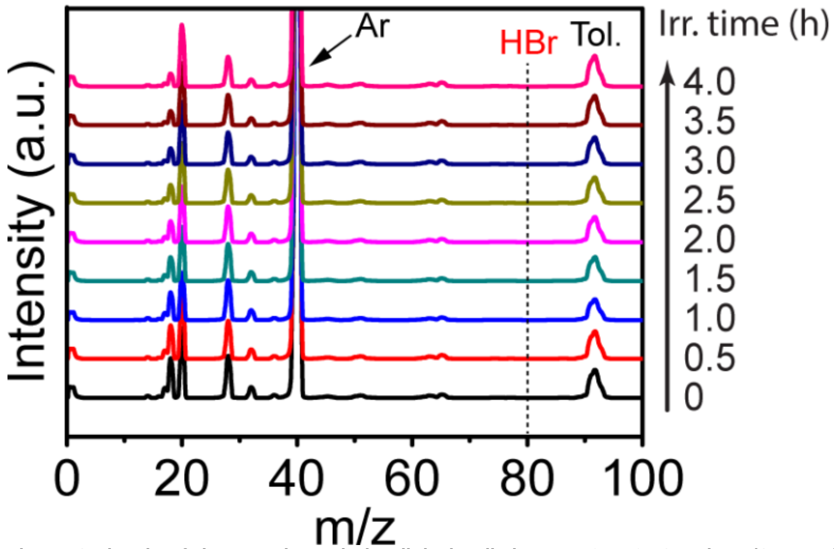

Figure S21. Time-dependent MS signals of the gas phase during light irradiation on 10 wt.% $Cs_3Bi_2Br_9$/SBA-15 in toluene with Ar flow bubbling.

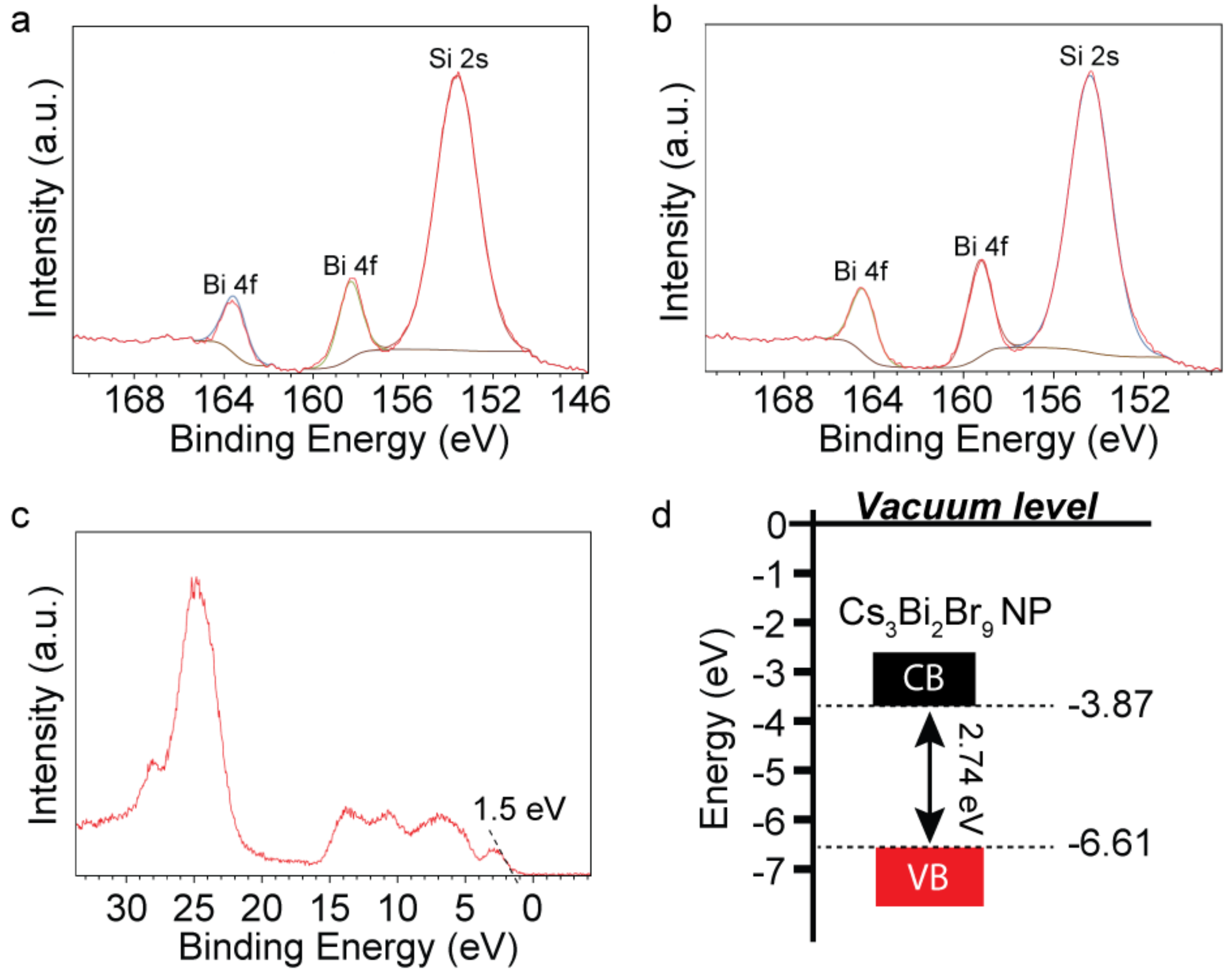

Figure S22. **XPS analysis and band positions.** High-resolution Bi 4f XPS spectra for **a,** the fresh and **b,** irradiated $Cs_3Bi_2Br_9$/SBA-15 samples. **c,** Valence band spectrum and **d,** band alignment of the fresh $Cs_3Bi_2Br_9$/SBA-15 sample (work function of 5.11 eV[21]).

Both yellow supported $Cs_3Bi_2Br_9$/SBA-15 sample and bulk $Cs_3Bi_2Br_9$ sample could turn to black after light irradiation in toluene under anaerobic conditions (Ar atmosphere, Figure S18). As the Bi oxidation state did not change (Figure S22a-b), we assumed that the colour change is due to two possible reasons. One relates to the hydron species on surface, which may come from the methyl group of toluene. This could be proved by the fact that a coupling product of 1,2-diphenylethane was generated as the main product in the liquid phase and no H_2 was detected in the gas phase during the whole reaction (time resolved MS signals in Figure S21), which means the occurrence of C-H bond cleavage and fixation of hydrogen species on catalyst surface. Besides, the addition of TEMPO in this case could preserve the catalyst colour with the production of TEMPO-H due to capture of reduction species (H^+/e^-) (MS data in Figure S19b)[22], suggesting the adsorption of hydrogen species as a key point for the colour change. Furthermore, several solid-state ^1H nuclear magnetic resonance spectroscopy (NMR) signals of the deep-colour bulk $Cs_3Bi_2Br_9$ sample (cleaned and dried under vacuum before NMR measurement) indicate the existence of hydrogen atoms on surface, which could also come from the residual organic compounds (like toluene). To avoid the serious trapping of organic species in pores of SBA-15, we did not use irradiated $Cs_3Bi_2Br_9$/SBA-15 sample for ^1H NMR analysis, which may result in undesired interference. However, the heating treatment of this deep-colour sample in air at 120 ^oC for 48 h could make it back to yellow and the re-measurement of solid-state ^1H NMR showed the disappearance of a broad resonance peak at 2.3 ppm (Figure S20), which may belong to the adsorbed hydrogen species. Unfortunately, it is difficult to identify the exact adsorption location (Bi or Br atoms) for adsorbed hydrogen species. Another possible reason for this colour change of the catalyst could be the release of Br atoms in the form of HBr. However, no acid species can be observed in the liquid phase based on a pH paper indicator and the online measurement of gas phase by a mass spectrometer did not detect HBr molecules (Figure S18 and 21). Besides, XPS results of deep-colour samples (supported $Cs_3Bi_2Br_9$/SBA-15 or bulk $Cs_3Bi_2Br_9$) show that the atomic composition of Cs/Bi/Br is almost the same with the fresh one. Therefore, the

adsorption of hydrogen species from toluene could be the possible reason for the colour change of the irradiated photocatalyst under Ar atmosphere.

Table S8. Kinetic isotope effect (KIE) determined by using deuterated toluene as probe[a].

Substrate	Photocatalyst/solvent	*k* (h^{-1})	k_H/k_D
C_6H_5-CH_3	10 wt.% $Cs_3Bi_2Br_9$/SBA-15 /Benzene	9.3×10^{-3}	4.4
C_6D_5-CD_3	10 wt.% $Cs_3Bi_2Br_9$/SBA-15 /Benzene	2.1×10^{-3}	

[a] General reaction conditions: 10 mg photocatalyst, 0.1 mmol substrate, 5 mL solvent, 1 bar air at 293 K under visible light (≥420 nm) irradiation for 10h. *k* was calculated based on the pseudo-first-order reaction kinetics. The KIE was determined at the beginning stages for the photo-oxidation of toluene (conversion <10%).

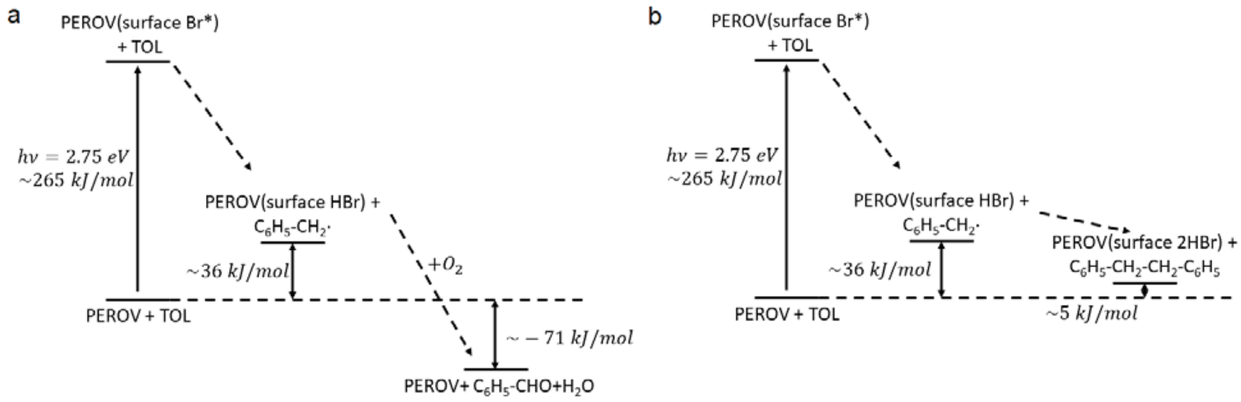

Figure S23. Thermochemistry analysis. The calculated potential energy of possible intermediates during toluene oxidation in aerobic (a) and anaerobic b) conditions, respectively (B3LYP-D3/def2-SVP level).

References

[1] D. Zhao, J. Feng, Q. Huo, N. Melosh, G. H. Fredrickson, B. F. Chmelka, G. D. Stucky, *Science* **1998**, *279*, 548-552.
[2] X. Deng, K. Chen, H. Tüysüz, *Chem. Mater.* **2017**, *29*, 40-52.
[3] B. Yang, J. Chen, F. Hong, X. Mao, K. Zheng, S. Yang, Y. Li, T. Pullerits, W. Deng, K. Han, *Angew. Chem. Int. Ed.* **2017**, *56*, 12471-12475.
[4] Y. Liu, L. Chen, Q. Yuan, J. He, C.-T. Au, S.-F. Yin, *Chem. Commun.* **2016**, *52*, 1274-1277.
[5] J. Zhang, F. Shi, J. Lin, D. Chen, J. Gao, Z. Huang, X. Ding, C. Tang, *Chem. Mater.* **2008**, *20*, 2937-2941.
[6] P. Edström, *J. Opt. Soc. Am. A* **2007**, *24*, 548-556.
[7] a)J. Shang, W. Hao, X. Lv, T. Wang, X. Wang, Y. Du, S. Dou, T. Xie, D. Wang, J. Wang, *ACS Catal.* **2014**, *4*, 954-961; b)J. Tauc, R. Grigorovici, A. Vancu, *Phys. Status Solidi B* **1966**, *15*, 627-637.
[8] J. Liu, Y. Liu, N. Liu, Y. Han, X. Zhang, H. Huang, Y. Lifshitz, S.-T. Lee, J. Zhong, Z. Kang, *Science* **2015**, *347*, 970-974.
[9] F. Neese, *Wiley Interdiscip. Rev. Comput. Mol. Sci.* **2012**, *2*, 73-78.
[10] a)V. M. García, R. Caballol, J. P. Malrieu, *J. Chem. Phys.* **1998**, *109*, 504-511; b)S. Grimme, S. Ehrlich, L. Goerigk, *J. Comput. Chem.* **2011**, *32*, 1456-1465; c)B. Metz, H. Stoll, M. Dolg, *J. Chem. Phys.* **2000**, *113*, 2563-2569; d)J. P. Perdew, K. Burke, M. Ernzerhof, *Phys. Rev. Lett.* **1996**, *77*, 3865-3868; e)F. Weigend, R. Ahlrichs, *Phys. Chem. Chem. Phys.* **2005**, *7*, 3297-3305.
[11] a)A. D. Becke, *J. Chem. Phys.* **1993**, *98*, 5648-5652; b)S. Grimme, J. Antony, S. Ehrlich, H. Krieg, *J. Chem. Phys.* **2010**, *132*, 154104; c)F. Weigend, *Phys. Chem. Chem. Phys.* **2006**, *8*, 1057-1065.
[12] a)F. Lazarini, *Acta Crystallogr., Sect. B: Struct. Sci* **1977**, *33*, 2961-2964; b)B. Yang, J. Chen, S. Yang, F. Hong, L. Sun, P. Han, T. Pullerits, W. Deng, K. Han, *Angew. Chem. Int. Ed.* **2018**, *57*, 5359-5363.
[13] S. J. Gregg, K. S. W. Sing, *Adsorption, Surface Area and Porosity*, Academic Press, New York, **1982**.
[14] Y. Dai, H. Tüysüz, *ChemSusChem* **2019**, *12*, 2587-2592.
[15] a)C. Hall, R. N. Perutz, *Chem. Rev.* **1996**, *96*, 3125-3146; b)J. A. Calladine, S. B. Duckett, M. W. George, S. L. Matthews, R. N. Perutz, O. Torres, K. Q. Vuong, *J. Am. Chem. Soc.* **2011**, *133*, 2303-2310.
[16] W. Pan, H. Wu, J. Luo, Z. Deng, C. Ge, C. Chen, X. Jiang, W.-J. Yin, G. Niu, L. Zhu, L. Yin, Y. Zhou, Q. Xie, X. Ke, M. Sui, J. Tang, *Nat. Photonics* **2017**, *11*, 726-732.
[17] a)X. Cao, Z. Chen, R. Lin, W.-C. Cheong, S. Liu, J. Zhang, Q. Peng, C. Chen, T. Han, X. Tong, Y. Wang, R. Shen, W. Zhu, D. Wang, Y. Li, *Nat. Catal.* **2018**, *1*, 704-710; b)H. Huang, H. Yuan, J. Zhao, G. Solís-Fernández, C. Zhou, J. W. Seo, J. Hendrix, E. Debroye, J. A. Steele, J. Hofkens, J. Long, M. B. J. Roeffaers, *ACS Energy Lett.* **2019**, *4*, 203-208; c)R. Yuan, S. Fan, H. Zhou, Z. Ding, S. Lin, Z. Li, Z. Zhang, C. Xu, L. Wu, X. Wang, X. Fu, *Angew. Chem. Int. Ed.* **2013**, *125*, 1069-1073; d)Y. Zhang, N. Zhang, Z.-R. Tang, Y.-J. Xu, *Chem. Sci.* **2012**, *3*, 2812-2822; e)L. Kesavan, R. Tiruvalam, M. H. A. Rahim, M. I. bin Saiman, D. I. Enache, R. L. Jenkins, N. Dimitratos, J. A. Lopez-Sanchez, S. H. Taylor, D. W. Knight, C. J. Kiely, G. J. Hutchings, *Science* **2011**, *331*, 195-199; f)M. I. bin Saiman, G. L. Brett, R. Tiruvalam, M. M. Forde, K. Sharples, A. Thetford, R. L. Jenkins, N. Dimitratos, J. A. Lopez-Sanchez, D. M. Murphy, D. Bethell, D. J. Willock, S. H. Taylor, D. W. Knight, C. J. Kiely, G. J. Hutchings, *Angew. Chem. Int. Ed.* **2012**, *51*, 5981-5985; g)X.-H. Li, X. Wang, M. Antonietti, *ACS Catal.* **2012**, *2*, 2082-2086; h)H. Wang, L. Wang, J. Zhang, C. Wang, Z. Liu, X. Gao, X. Meng, S. J. Yoo, J.-G. Kim, W. Zhang, F.-S. Xiao, *ChemSusChem* **2018**, *11*, 3965-3974.
[18] Y.-R. Luo, Ed., *Handbook of Bond Dissociation Energies in Organic Compounds; CRC Press: London, U.K.,* **2003**.
[19] P.-C. Nam, M. T. Nguyen, A. K. Chandra, *J. Phys. Chem. A* **2005**, *109*, 10342-10347.
[20] M. Krasowska, W. B. Schneider, M. Mehring, A. A. Auer, *Chem. Eur. J.* **2018**, *24*, 10238-10245.
[21] X. Zhu, Y. Lin, Y. Sun, M. C. Beard, Y. Yan, *J. Am. Chem. Soc.* **2019**, *141*, 733-738.
[22] R. Amorati, G. F. Pedulli, D. A. Pratt, L. Valgimigli, *Chem. Commun.* **2010**, *46*, 5139-5141.
